# Molecular Simulation of Interfacial Chemistry of Oxygen and Water Molecules Within Defective Graphene/MoS_2_ Heterojunctions

**DOI:** 10.3390/nano16181173

**Published:** 2026-09-17

**Authors:** Xu Zhang, Suang Li, Xiaoning Yang

**Affiliations:** 1 State Key Laboratory of Materials-Oriented Chemical Engineering, College of Chemical Engineering, Nanjing Tech University, Nanjing 211816, China; zhangxu@njtech.edu.cn (X.Z.); egbertchang@outlook.com (S.L.); 2 Information Construction Center, Nanjing Tech University, Nanjing 211816, China

**Keywords:** heterojunction, graphene, MoS_2_, interlayer chemistry, molecular simulation

## Abstract

The graphene/MoS_2_ composite is a functional heterostructure with broad applications that leverage the synergistic properties of its constituents. Generally, small ambient molecules might penetrate and adsorb into the interlayer of graphene/MoS_2_ and interact with surface defects, leading to chemical reactions within the confined spaces. Currently, the interfacial interaction and reaction mechanisms remain largely unclear. Herein, we employed density functional theory and ab initio molecular dynamics simulations to investigate the interfacial adsorptions and reactions of O_2_ and H_2_O molecules at the Graphene/MoS_2_ interfaces with various vacancy types of MoS_2_ surfaces. Various mechanisms and pathways have been identified for the thermodynamic trends and kinetic barriers in interlayer reactions. It is demonstrated that the pristine Graphene/MoS_2_ is inert for molecular interlayer adsorption. However, there are obvious chemical reactions at the defect-involved G/MoS_2_ interfaces. Specifically, the dissociation of molecular oxygen can induce surface oxidation, accompanied by the restoration of the electronic properties of graphene/MoS_2_. The water molecule can undergo spontaneous dissociation at the defect sites, exhibiting enhanced activity for water splitting. As compared with bare MoS_2_, the hybrid interfaces of graphene/MoS_2_ can alter the chemical reactivities of adsorbed molecules. This work provides new insights into the interlayer chemistry for heterojunction interfaces.

## 1. Introduction

Currently, monolayer graphene (G) can be interfaced with layered molybdenum disulfide (MoS_2_) crystals through the vdW interaction to form G/MoS_2_ heterojunction composites or hybrid structures, harnessing their synergistic effects for novel applications. For example, G/MoS_2_ exhibits excellent performance as a photocatalyst for degrading organic pollutants [1], and meanwhile, it can enhance energy and power densities as an electrocatalyst [2,3]. In electronic devices, G/MoS_2_ also acts as a high-performance field-effect transistor [4]. As a prototypical layered material, MoS_2_ is commonly synthesized via chemical vapor deposition (CVD) [5] or mechanical exfoliation [6]. However, synthesis processes inevitably introduce intrinsic defects into the material surface [7], such as point defects, grain boundaries, and antisite defects. Experimental studies have identified various types of MoS_2_ surface vacancies, including S vacancies (VS) [8], S di-vacancies (VS_2_) [9], and MoS_2_ triple vacancies (VMoS_2_) [10]. In addition to intrinsic defects, engineered defects can be artificially introduced into MoS_2_ to tailor its properties for specific applications [11].

Defects can modify the physical and electronic properties of MoS_2_, which in turn dictate the performance of MoS_2_-based heterostructures. For instance, defects in G/MoS_2_ heterojunction systems can suppress electron transport, induce charge trapping, and cause Fermi-level pinning as well as a finite Schottky barrier [12], which reduces the performance of field-effect transistor architectures [13]. Moreover, it has also been shown that defects can undermine the electrical properties of G/MoS_2_ by reducing carrier mobility and hindering electron transport through localization [14].

During fabrication, transfer, and exposure to humid conditions, small ambient molecules (e.g., O_2_ and H_2_O) inevitably intercalate into the interlayer of G/MoS_2_ [15,16,17], which might alter the structural and electronic properties. A previous study [18] has observed that the interaction between O_2_ and the MoS_2_/Si heterojunction can cause oxidation, leading to a decline in solar cell lifespan. While it has been demonstrated that pristine graphene or MoS_2_ exhibits relative inertness to O_2_ and H_2_O molecules [19,20], defect sites can interact with these molecules, forming molybdenum oxides (MoO_x_) [21] and graphene oxide [22], which degrade electronic devices. On the contrary, in certain cases, defects can be repaired through the chemical adsorption of water [23] and oxygen [24], thereby enhancing the durability of the material. Moreover, the graphene cover may influence molecular reaction behavior at the interlayer, as a graphene monolayer can induce the “catalysis under cover” effect with the substrate [17]. Hence, a comprehensive understanding of the interlayer reaction mechanisms of O_2_ and H_2_O in G/MoS_2_ is vital for elucidating the oxidation-driven performance degradation and the defect repair process, thus improving the application of G/MoS_2_ composites.

Although the reaction mechanisms of bare graphene [25] and MoS_2_ [19] have been studied, the interactions of O_2_ and H_2_O molecules with the G/MoS_2_ interlayer have been less investigated so far. It is highly important to understand how the small molecules can interact, react, or even modify the interfaces. Currently, the complexity of the interfacial reaction processes poses challenges for direct experimental characterization. Comparatively, molecular simulation could offer a viable approach for probing atomic-scale interactions and reaction mechanisms. Nevertheless, existing simulation studies mainly focus on thermal transport [26], optical properties [27], and energy storage [28], with limited attention given to its chemical reactivity and interlayer oxidation behavior. In addition, the interactions of defects with O_2_ or H_2_O may release reactive O and H species [19,29]. Thus, characterizing the diffusion behavior of free O and H atoms within the G/MoS_2_ interlayer is also important for understanding the interlayer reactivity.

In this study, we investigated the reactivity of O_2_ and H_2_O on the pristine G/MoS_2_ interface, as well as on the G/MoS_2_ interfaces containing various vacancy types on the MoS_2_ surfaces, such as G/VS, G/VS_2_, and G/VMoS_2_. We employed ab initio density functional theory (DFT) simulations with the transition state search method to examine the chemical reactions of O_2_ and H_2_O molecules at the interface between monolayer graphene and MoS_2_ surfaces. Compared to the bare MoS_2_ surface, different reaction mechanisms and pathways were proposed and analyzed for the defected G/MoS_2_ heterostructures. Meanwhile, ab initio molecular dynamics (AIMD) simulations were applied to examine the relevant dynamical reaction processes. The simulation work discloses the impact of the interlayer reactions on the electronic properties of the G/MoS_2_ heterojunction, and more importantly, it offers key insights into surface oxidation, defect repair, and water splitting, thereby providing the technological applications of G/MoS_2_ composites.

## 2. Materials and Methods

In this work, DFT calculations were conducted using the spin-polarized method with the periodic materials module (BAND) of the Amsterdam Density Functional (ADF 2019.306) program [30]. Geometry optimizations and total energy calculations were performed using the generalized gradient approximation (GGA) [31] with the Perdew-Burke-Ernzerhof (PBE) functional [32] and the D3 dispersion correction [33]. This method is extensively used to describe van der Waals interactions in layered materials [13,34]. For calculations involving the band structure and partial density of states (PDOS), we utilized the Heyd-Scuseria-Ernzerhof (HSE06) hybrid functional [35], which can provide reduced band gap errors in MoS_2_ compounds [36]. A detailed justification of computational methods is provided in the Supplementary Materials. The Brillouin zone was sampled by a (6 × 6 × 1) k-point mesh. The Slater-type triple-Z atomic orbital with one polarization function (TZP) was utilized in all calculations [37]. The transition state search method with the climbing image nudged elastic band (CI-NEB) [38] method was used for reaction and diffusion pathway analysis.

AIMD simulations were performed using the CP2K 7.1 package [39]. Molecular dynamics simulations were carried out in the canonical (NVT) ensemble, utilizing the Nosé-Hoover thermostat for temperature control [40]. The temperature was set to 1000 K with a time step of 1.0 fs. The high temperature was employed to enhance the sampling of reactive events within the limited timescale accessible to AIMD simulations [29,41]. The high-temperature AIMD simulation can provide support for the proposed reaction pathways. The plane-wave cutoff was specified as 400 Ry, and the convergence threshold was set to 1.0 × 10^−5^.

The adsorption energy of the adsorbate was calculated as:

(1) $$E_{ads}=\left[E_{\left(system\right)}-\left(E_{\left(G/X\right)}+E_{\left(a\right)}\right)\right],$$

where $E_{\left(system\right)}$, $E_{\left(G/X\right)}$, and $E_{\left(a\right)}$ refer to the total energies of the system with adsorbate, the system without adsorbate, and the isolated adsorbate, respectively. X represents MoS_2_, which includes different defects: VS, VS_2_, and VMoS_2_. To enhance the accuracy of adsorption energy calculations, the basis set superposition error (BSSE) correction [42] was applied.

In this work, the simulation cell consisted of a 4 × 4 MoS_2_ supercell and a 5 × 5 graphene supercell, ensuring minimal lattice mismatch. Herein, four heterojunction configurations were considered, including the G/MoS_2_, G/VS, G/VS_2_, and G/VMoS_2_ systems. Figure S1a presents the optimized pristine G/MoS_2_ system (top and side views), which exhibits an interlayer distance of 3.39 Å. The corresponding band structure (Figure S1b) reveals a small band gap of 13 meV. These results agree well with the data reported in previous studies [43,44]. The calculated binding energy of −1.4 eV is consistent with the previous report [45], further validating the computational reliability and confirming the structural stability of this model. Figure S1c shows all the optimized defective structures, which were generated by selectively removing specific atoms from the pristine G/MoS_2_ system. Specifically, one S atom was removed to form G/VS, two adjacent S atoms were removed to form G/VS_2_, and a Mo atom and two adjacent S atoms were removed to form G/VMoS_2_. The calculated binding energies of −2.43 eV for G/VS, −2.75 eV for G/VS_2_, and −2.69 eV for G/VMoS_2_ suggest that the defective heterojunctions still exhibit good structural stability.

## 3. Results and Discussion

### 3.1. Adsorption of O_2_ and H_2_O at G/MoS_2_ Interface

**Figure 1:**
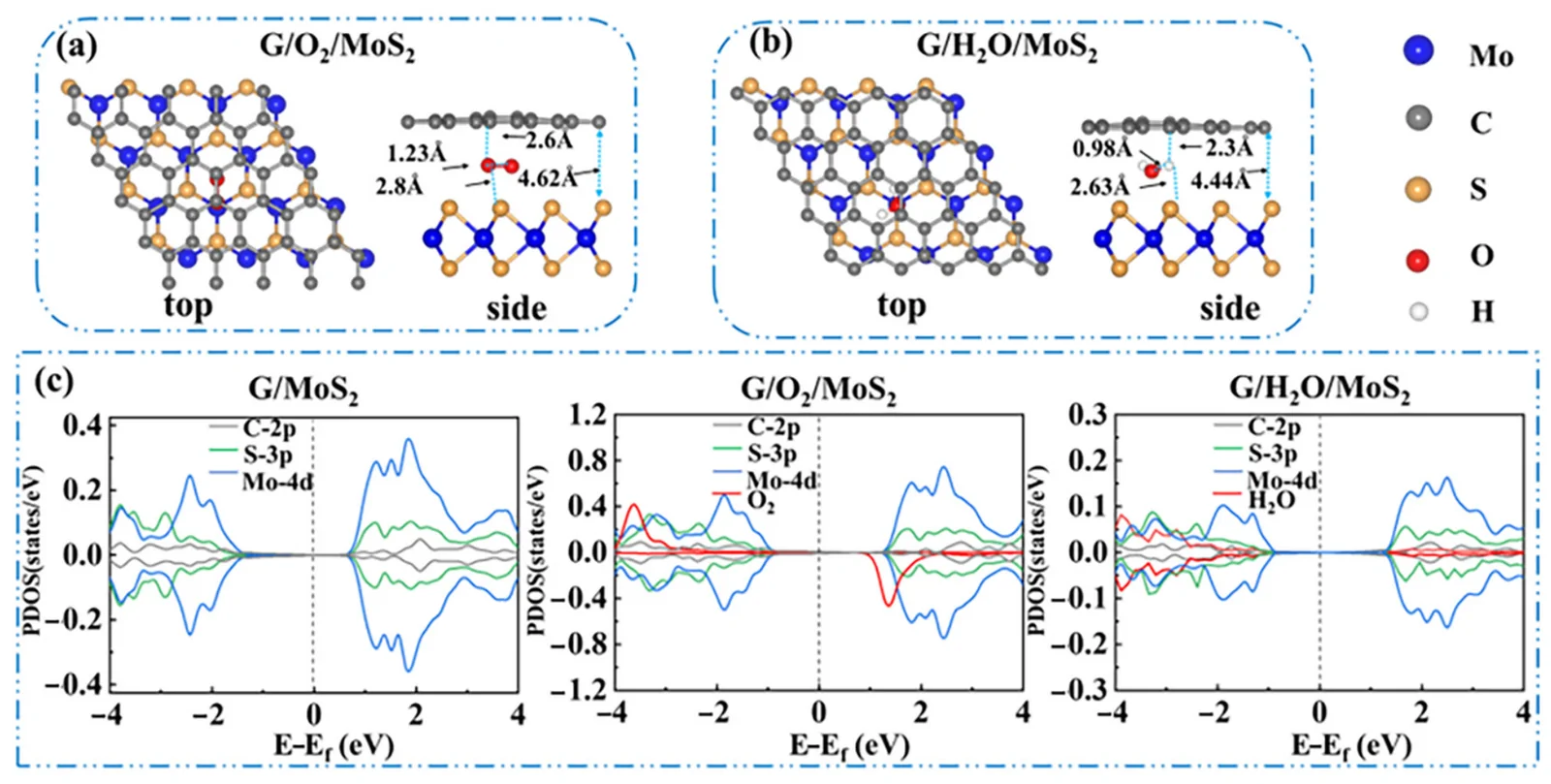
Top and side views of the optimized G/O_2_/MoS_2_ (**a**) and G/H_2_O/MoS_2_ (**b**) systems. The blue dashed lines in the interlayer indicate the distances between two atoms. (**c**) The projected density of states of the G/MoS_2_, G/O_2_/MoS_2_, and G/H_2_O/MoS_2_ systems. The Fermi level is set to zero and shown by a gray dashed line.

We first examined the adsorption interaction of a single O_2_/H_2_O molecule within the pristine G/MoS_2_ interface, referred to as G/O_2_/MoS_2_ (Figure 1a) and G/H_2_O/MoS_2_ (Figure 1b), respectively. The interlayer spacings are ~4.62 Å in G/O_2_/MoS_2_ and ~4.44 Å in G/H_2_O/MoS_2_, which are larger than that in G/MoS_2_ (~3.39 Å). This indicates that the intercalation of O_2_ or H_2_O weakens the interlayer interactions. The adsorption energies ($∆E_{ads}$) of O_2_ (0.4 eV) and H_2_O (0.25 eV) at the G/MoS_2_ interface further suggest that molecular intercalation is implausible. A similar phenomenon has been observed for O_2_ entering the interlayer of the G/TiO_2_ system [16]. The transition state search method was employed to analyze the possible reaction processes during O_2_ and H_2_O interlayer adsorption. Figure S2 shows the energy profiles and the critical configurations along the possible pathways. The higher activation barriers and thermodynamic disadvantage for the dissociation of O_2_ (Figure S2a) and H_2_O (Figure S2b) suggest that the chemical processes are unfavorable. Furthermore, the projected density of states (PDOS) (Figure 1c) of G/O_2_/MoS_2_ and G/H_2_O/MoS_2_ is nearly identical to that of pristine G/MoS_2_. These results indicate that H_2_O and O_2_ hardly interact with the G/MoS_2_ interface. This can be attributed to the inherent chemical inertness of both graphene [46] and MoS_2_ [47]. Overall, the G/MoS_2_ interlayer space is unlikely to allow chemical adsorption of molecules.

### 3.2. O_2_ at G/VS Interface

**Figure 2:**
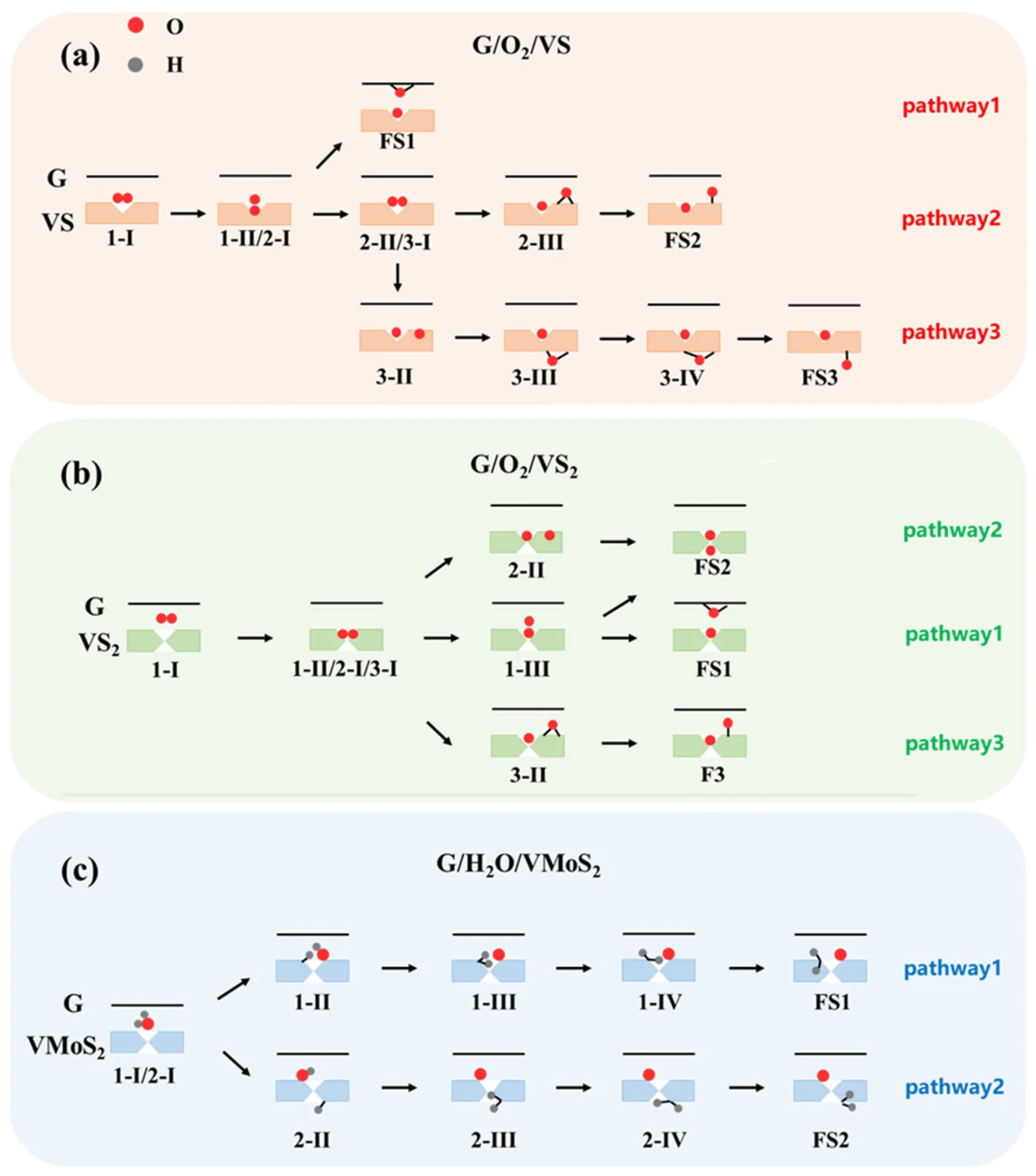
Schematic reaction pathways of ambient molecules (O_2_ or H_2_O) at the heterojunction interfaces. O_2_ adsorption at the G/VS (**a**) and G/VS_2_ (**b**) interfaces follows three different reaction pathways. (**c**) H_2_O adsorption at the G/VMoS_2_ interface exhibits two different reaction pathways. Hydrogen atoms are marked in gray circles, oxygen in red circles, and graphene in black lines. The orange, green, and blue rectangles represent VS, VS_2_, and VMoS_2_, respectively.

At the G/VS interface with the surface vacancy, molecular oxygen might penetrate the interlayer and interact with the vacancy atoms [17,48]. The reactivity of O_2_ can be proposed to follow three reaction pathways at the G/VS interface (Figure 2a). The corresponding final states of pathways 1, 2, and 3 are hereafter denoted as FS1, FS2, and FS3, respectively. Pathway 1 begins with the adsorption of O_2_ (1-I) near the S vacancy on the G/VS interface, followed by a transition to an upright configuration (1-II). Afterward, the O_2_ molecule dissociates and occupies the S vacancy while concurrently inducing the oxidation of graphene (FS1). Pathway 2 involves the transition of O_2_ from an upright (2-I) to a lying-down configuration (2-II). After dissociation, the S vacancy is occupied while simultaneously forming a Mo-O-S bond, which eventually forms an S-O bond (FS2). Pathway 3 offers an alternative lying-down (3-I) adsorption mechanism, where one O atom occupies the S vacancy while the other O atom diffuses into the Mo plane (3-II). Ultimately, an S-O bond is formed at the outer layer (FS3).

**Figure 3:**
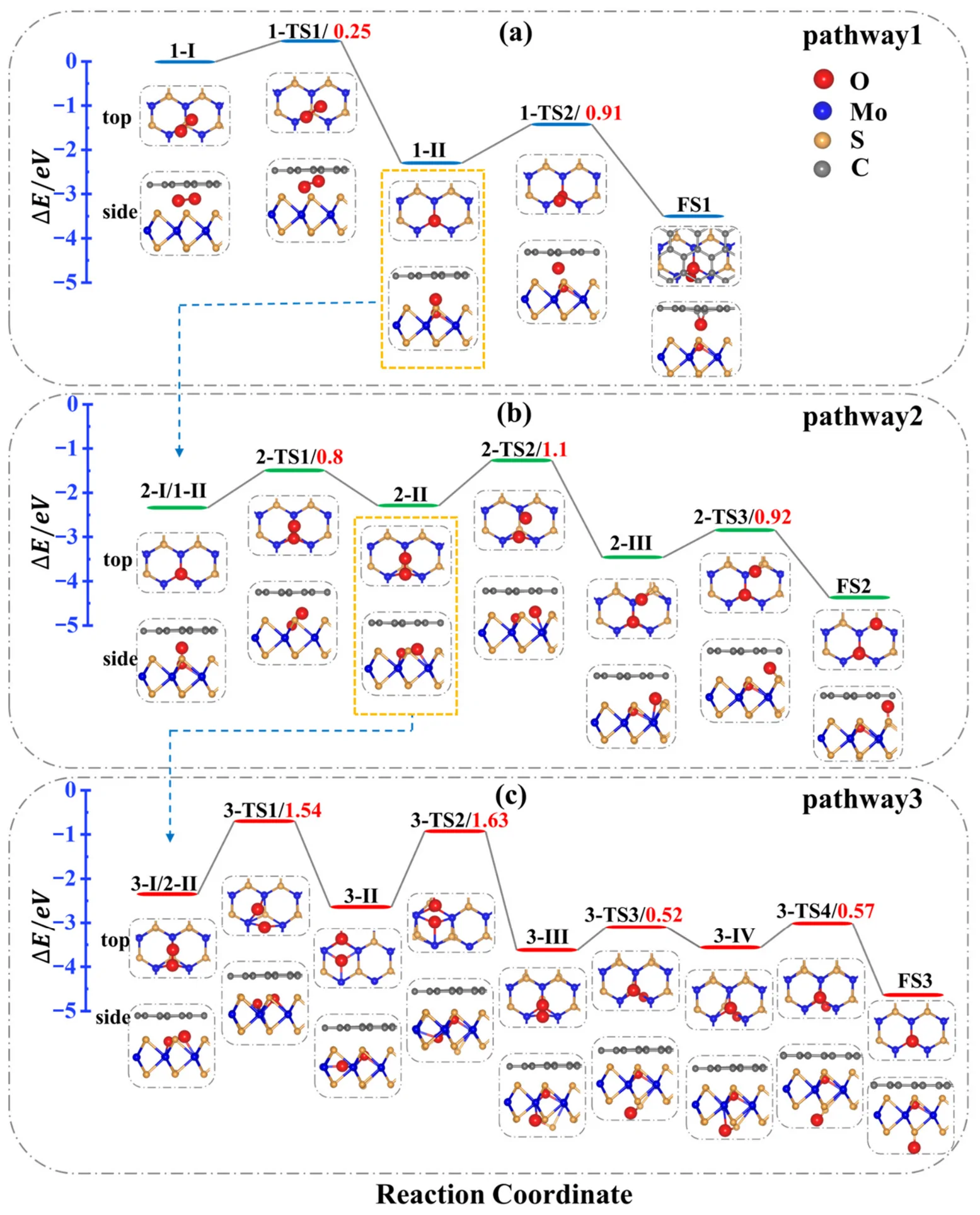
Calculated energy profiles of the reaction pathways of O_2_ at the G/VS interface, accompanied by the corresponding structural information (top and side views): (**a**) pathway1; (**b**) pathway2; (**c**) pathway3. The reaction pathway illustrates the transition state (TS) and key intermediates of the elementary reaction, with activation barrier values indicated in red.

Figure 3 presents the energy profile curves for the above reaction pathways, along with the critical configurations. Tables S1–S3 present the relevant bonding topological parameters. In pathway 1, the initial structure (1-I) is mainly generated through the physical interaction between O_2_ and the G/VS interface (Figure S3a, G/O_2_/VS). The adsorption of O_2_ on the G/VS interface is more stable than that on the G/MoS_2_ interface, with an $∆E_{ads}$ of −0.22 eV. This is likely because the S vacancy provides an adsorption site [49] and reduces the interlayer spacing (3.74 Å), thus enhancing the adsorption interaction. As expected, O_2_ is more strongly adsorbed on the G/VS interface than on the bare vs. surface [19], denoting that graphene can stabilize the interlayer adsorption. The further reaction generates an upright state of active oxygen (1-II) species and releases an energy of 2.33 eV with a low activation barrier (1-TS1, 0.25 eV). One O atom in O_2_ can form ionic bonds with three Mo atoms and create the MoO_x_ (Table S1). The activation barrier is close to the reported values (0.19–0.35 eV) for the reaction between O_2_ and the bare monolayer vs. surface [48,50,51], indicating that the process readily occurs under mild conditions.

The O_2_ molecule subsequently dissociates into two O atoms: One O atom occupies the S vacancy, forming the MoO_x_ with Mo-O ionic bonds (Table S1) on the MoS_2_ surface. This oxidation behavior has also been observed experimentally [52]. The other O atom forms an epoxide on the graphene surface. The reaction is typically as follows: G/VS + O_2_ → VS-O + G-O, which exhibits a moderate activation barrier (1-TS2, 0.91 eV) and an energy drop of −1.35 eV. It can be seen that the graphene cover alters the O_2_ oxidation pathway on MoS_2_ [19,51], potentially changing the structural and electronic properties of the G/MoS_2_ heterojunction.

In pathway 2 (Figure 3b), the out-of-plane O atom might bond with the Mo atom through an ionic bond (Table S2), transitioning to a lying-down configuration (2-II) with an activation barrier of 0.8 eV (2-TS1). This is attributed to the smaller size of the O atom, which makes it more likely to interact with Mo atoms. In the following process, one O atom occupies the S vacancy, while another O atom forms a Mo-O-S bond (2-II). Then the Mo-O bond breaks, creating an S-O ionic bond (Table S2). The activation process is represented by 2-TS2 (1.1 eV) and 2-TS3 (0.92 eV), both of which can be overcome under high-temperature conditions [21]. This reaction mechanism agrees with the reported reaction behavior of O_2_ on the vs. surface in the previous literature [50,51]. However, our result for the G/VS interface shows a higher activation barrier. This result suggests that graphene might provide some degree of “protection” for the substrate from experiencing oxidation [53]. At this stage, the desorption of SO species would need to overcome a higher energy barrier (3.9 eV), which renders the desorption procedure infeasible. Our simulation shows that O_2_ adsorption can occupy the S vacancy, in agreement with the observed behavior in oxygen-assisted growth [54] and oxygen substitution on monolayer MoS_2_ [55]. As shown in Figure 3a,b, in pathways 1 and 2, the overall net reaction processes for molecular oxygen at the G/VS interface are energetically favorable. Meanwhile, the small difference in activation barriers between pathways 1 and 2 suggests that both processes can occur concurrently.

In pathway 3 (Figure 3c), the activation barrier for the O_2_ dissociation (3-TS1, 1.54 eV) is significantly higher than in other pathways. This can be attributed to the weak interaction between the O atom and the Mo atom far from the vacancy (Table S3). Afterward, the O atom in the plane of Mo atoms forms an S-O bond with the bottom S atom of MoS_2_. This exothermic process might occur in multiple steps, with activation barriers of 1.63 eV (3-TS2), 0.52 eV (3-TS3), and 0.57 eV (3-TS4) for each step. Compared to pathways 1 and 2, pathway 3 has a higher energy barrier with multiple reaction steps, making the reaction dynamics less favorable.

**Figure 4:**
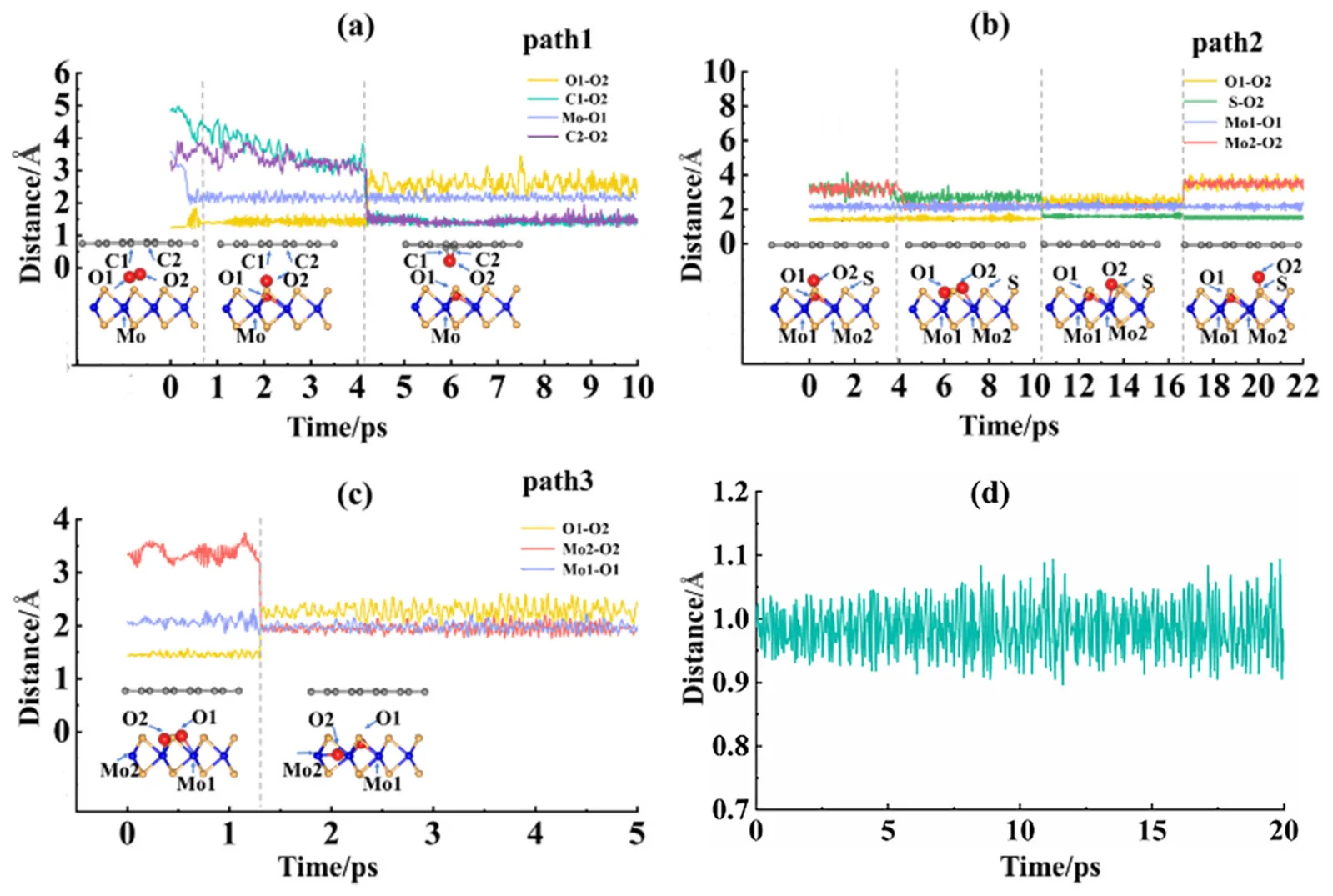
Time evolution of interatomic distances and corresponding structural changes obtained from AIMD simulations. (**a**–**c**) present the simulation results of pathway 1, pathway 2, and pathway 3 for the O_2_ reaction at the G/VS interface, respectively. (**d**) The variation in S-O bond length in the FS3 state. The different interatomic distances are represented as follows: O-O (orange curve), Mo-O (mauve and pink curves), C-O (purple and light green curves), and S-O (green curve).

Next, AIMD simulations were conducted to directly examine the dynamical reaction process of O_2_ at the G/VS interface for the above three pathways. Figure 4 illustrates the time evolution of several typical interatomic distances in the G/O_2_/VS system, together with the corresponding structural changes. Figure 4a shows that O_2_ can interact with the S vacancy to form Mo-O bonds (~2.1 Å). Then, the O-O bonds split at ~4.25 ps, forming C-O bonds (~1.45 Å). This dynamical reaction supports the reaction mechanism in pathway 1 (Figure 3a) that the adsorbed O_2_ molecule can dissociate to form the Mo-O bond and realize the oxidation of graphene.

As shown in Figure 4b, the Mo2-O2 bond can be further changed from 3.3 Å to 2 Å, indicating a transition of O_2_ from an upright to a lying-down state. At 10.36 ps, O_2_ dissociates to occupy the vacancy and form a Mo-O-S bond (Mo-O bond ~2.1 Å, O-S bond ~1.6 Å), which ultimately breaks to generate an S-O bond (~1.5 Å). This dynamical reaction behavior also agrees with the formation of an S-O bond in pathway 2 (Figure 3b). This state was further simulated for an additional 20 ps (Figure 4d), during which no SO species were observed owing to the high desorption energy. However, as observed in Figure 4c, only the intermediate state (3-II) was observed during the AIMD simulation. This can be attributed to the observed high energy barrier shown in Figure 3c, which makes the whole reaction in pathway 3 unlikely to occur. The AIMD simulations provide support for the proposed reaction pathways and mechanisms for O_2_ at the G/VS interface.

**Figure 5:**
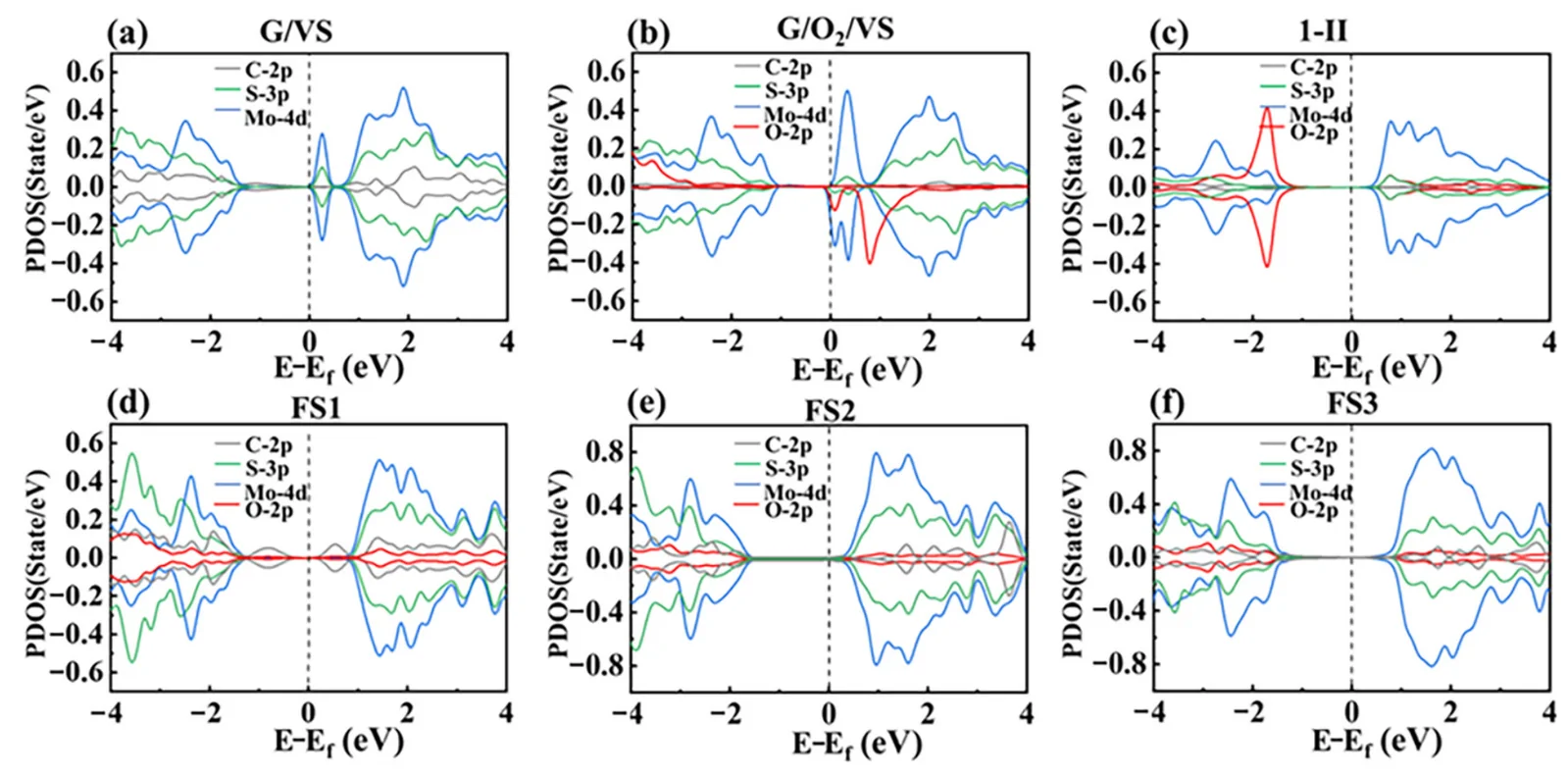
The PDOS results for the G/VS systems before and after O_2_ adsorption. (**a**) without O_2_ adsorption; (**b**) O_2_ physisorption; (**c**) O_2_ chemisorption; (**d**) FS1 state; (**e**) FS2 state; (**f**) FS3 state. Line color code: C-2p orbital, gray; S-3p orbital, green; Mo-4d orbital, blue; O-2p orbital, red. The Fermi level is set to zero and shown by a gray dashed line.

Figure 5 shows the PDOS results for the G/VS systems before and after the O_2_ adsorption. As shown in Figure 5a, the G/VS system is non-spin-polarized with defect peaks at the Fermi level, primarily originating from the 4d orbitals of Mo atoms and 3p orbitals of S atoms near the S vacancy [56]. These defects can enhance surface chemical reactivity [57]. Upon O_2_ physisorption (Figure 5b), the defect peak intensifies, disrupting symmetry and inducing spin polarization, demonstrating high sensitivity of the defects to O_2_. We note that the O_2_ chemisorption (Figure 5c) eliminates the defect peak, restoring symmetry and making the electronic structure identical to that of pristine G/MoS_2_ (Figure 1c). This means that O_2_ adsorption has a healing effect. PDOS analysis at the FS1 state (Figure 5d) shows small peaks near the Fermi level due to epoxy groups, with the bandgap increasing to 0.59 eV. This indicates that the electronic properties of the heterojunction have been modified, in which the semiconductor nature could be reestablished.

For the FS2 and FS3 states (Figure 5e,f), the presence of S-O bonds without new impurity peaks implies that the surface has been passivated. These PDOS are almost similar to those of pristine G/MoS_2_ with no significant change in the bandgap, further confirming that O_2_ can repair defects. Previous studies have only demonstrated that the presence of molecular oxygen can cause MoS_2_ degradation [19,58]. However, our study instead shows that O_2_ can repair the S vacancy, recovering its electronic properties. Similar findings of O_2_ defect repair have been reported in the SiO_2_/4H-SiC heterojunction [59]. Overall, the O_2_ molecule on the G/VS system not only causes surface oxidation but also repairs defects and restores its electronic properties.

### 3.3. O_2_ at G/VS_2_ Interface

In Figure 2b, three possible reaction pathways for O_2_ at the G/VS_2_ interface are proposed: Pathway 1 begins with the adsorption of O_2_ (1-I) near the interlayer S_2_ di-vacancy. Next, O_2_ undergoes two types of chemical adsorption (1-II → 1-III) within the interlayer. After dissociation, one O atom occupies a S vacancy, while the other O atom forms an epoxide with graphene (FS1). Alternatively, both O atoms may occupy the two S vacancies (FS2). Pathway 2 involves the dissociation of O_2_ from the lying-down state (2-I). One O atom occupies a S vacancy, while the other is initially located on the Mo plane (2-II) and subsequently occupies another S vacancy (FS2). In pathway 3, with O_2_ dissociating, one O atom occupies a S vacancy, while the other O atom forms a Mo-O-S bond (3-II). Ultimately, the Mo-O bond breaks and forms an S-O bond (FS3).

**Figure 6:**
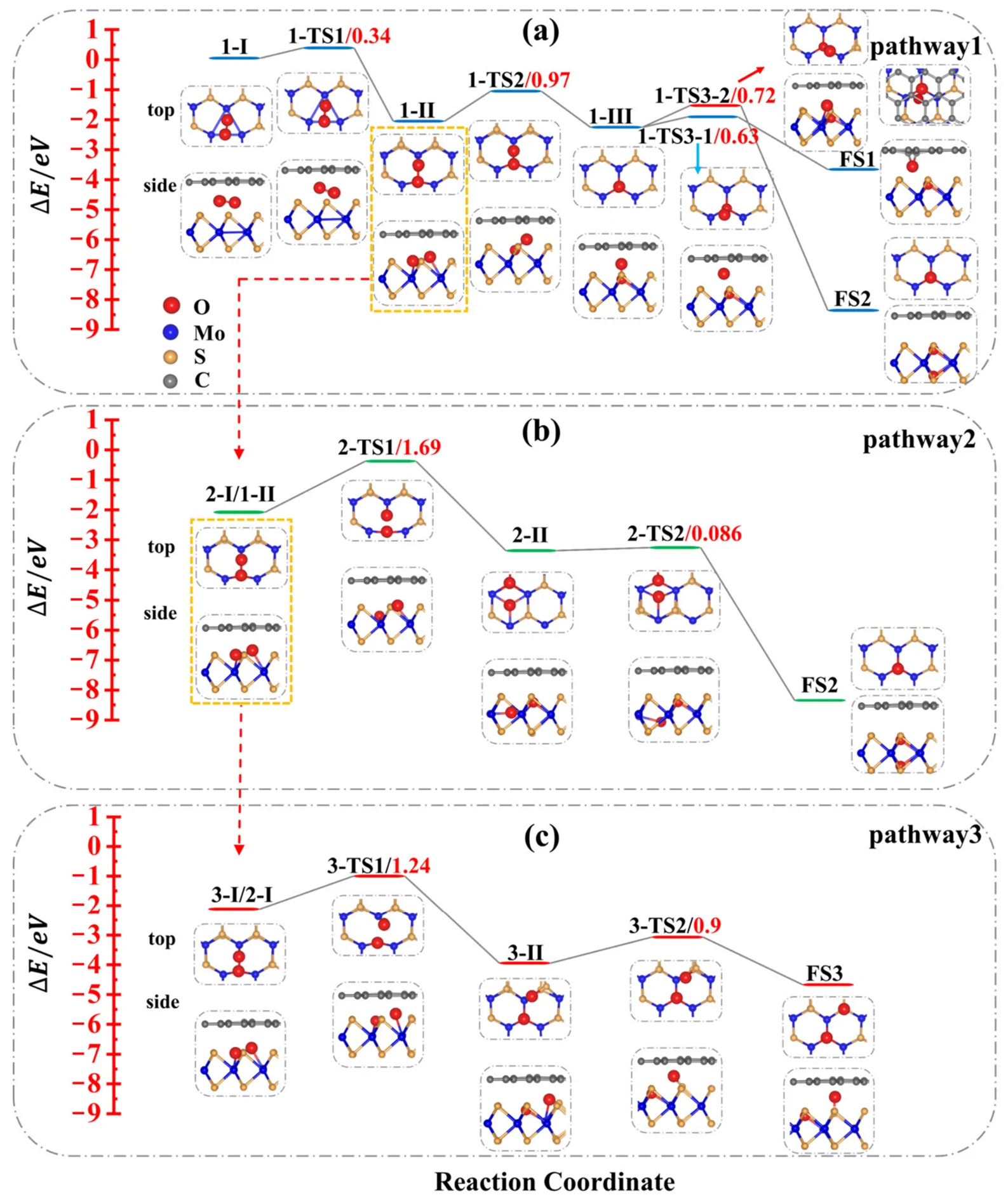
Calculated energy profiles of the reaction pathway of O_2_ at the G/VS_2_ interface, accompanied by the corresponding structural information (top and side views). (**a**) pathway1; (**b**) pathway2; (**c**) pathway3. The reaction pathway illustrates the transition state (TS) and key intermediates of the elementary reaction, with activation barrier values indicated in red.

The energetic profiles for the above reaction pathways of O_2_ at the G/VS_2_ interface are given in Figure 6. In pathway 1 (Figure 6a), the reaction process begins with the physical adsorption of O_2_ at the vacancy sites (G/O_2_/VS_2_, Figure S3b). The $∆E_{ads}$ of O_2_ on the G/VS_2_ interface (−0.28 eV) is slightly more negative than that on the G/VS interface. The further reaction shows that adsorbed O_2_ is activated through the elongation of the O-O bond with an activation barrier (1-TS1, 0.34 eV), eventually leading to the exothermic formation of the lying-down state (1-II). Similarly to the G/VS system, O_2_ can easily react at the S di-vacancy, wherein the two O atoms form Mo-O ionic bonds with the Mo atoms. Detailed bonding information has been provided in Tables S4–S6. Next, after surpassing a barrier of 0.97 eV (1-TS2), the O_2_ transfers into an upright state (1-III). Although the two chemical adsorption states (upright and lying-down states) of O_2_ on the monolayer VS_2_ surface have been individually identified in previous studies [19,51], the transition between the two states in the G/VS_2_ system is first reported here. This behavior can be attributed to the larger vacancy at the G/VS_2_ interface, which provides sufficient space for O_2_ to adopt different configurations.

At the 1-III stage, the dissociation of O_2_ is accompanied by two possible processes. In the first process, one O atom occupies a S vacancy. The second O atom forms an epoxide with graphene (FS1). This reaction has an activation barrier of 0.63 eV (1-TS3-1), releasing the reaction energy of 1.35 eV. Unlike pathway 1 at the G/VS interface (Figure 3a), this pathway retains a vacancy, which may induce further oxidation. However, the second process shows that the two vacancies can be occupied by an O radical pair with an activation barrier of 0.72 eV (1-TS3-2), forming Mo-O ionic bonds (FS2). AIM analysis (Table S4) for the 1-TS3-2 state shows the bonding information between the dissociated O atom and the Mo atom. The activation energy barrier for this reaction is higher than that of bare VS_2_ [51], indicating that the graphene cover influences the oxidation reaction.

For pathway 2, the adsorbed O_2_ molecule in the lying-down state (2-I) dissociates via two procedures: one O atom occupies a S vacancy to form Mo-O bonds (Table S5), and the second O atom diffuses into the Mo plane (2-II), involving a higher energy barrier of 1.69 eV (2-TS1). Eventually, the O radical pair occupies two S vacancies with a lower activation barrier (2-TS2, 0.086 eV). In pathway 3 (Figure 6c), the O_2_ dissociation follows a similar reaction mechanism to pathway 2 in the G/VS system (Figure 3b) with a comparable activation barrier. That is, one O atom occupies the S vacancy, and the other O atom forms a Mo-O-S bond (3-II). Ultimately, the O atom in the Mo-O-S bond moves to the top of the S atom, forming an S-O ionic bond (Table S6). The two steps involve activation barriers of 1.24 eV (3-TS1) and 0.9 eV (3-TS2). Comparatively, they are higher than those for O_2_ dissociation on the bare VS_2_ surface [56]. Likewise, the desorption of SO gas is unlikely to occur. Notably, a vacancy still exists in the FS3 state, which may affect the long-term performance of the heterojunction material.

**Figure 7:**
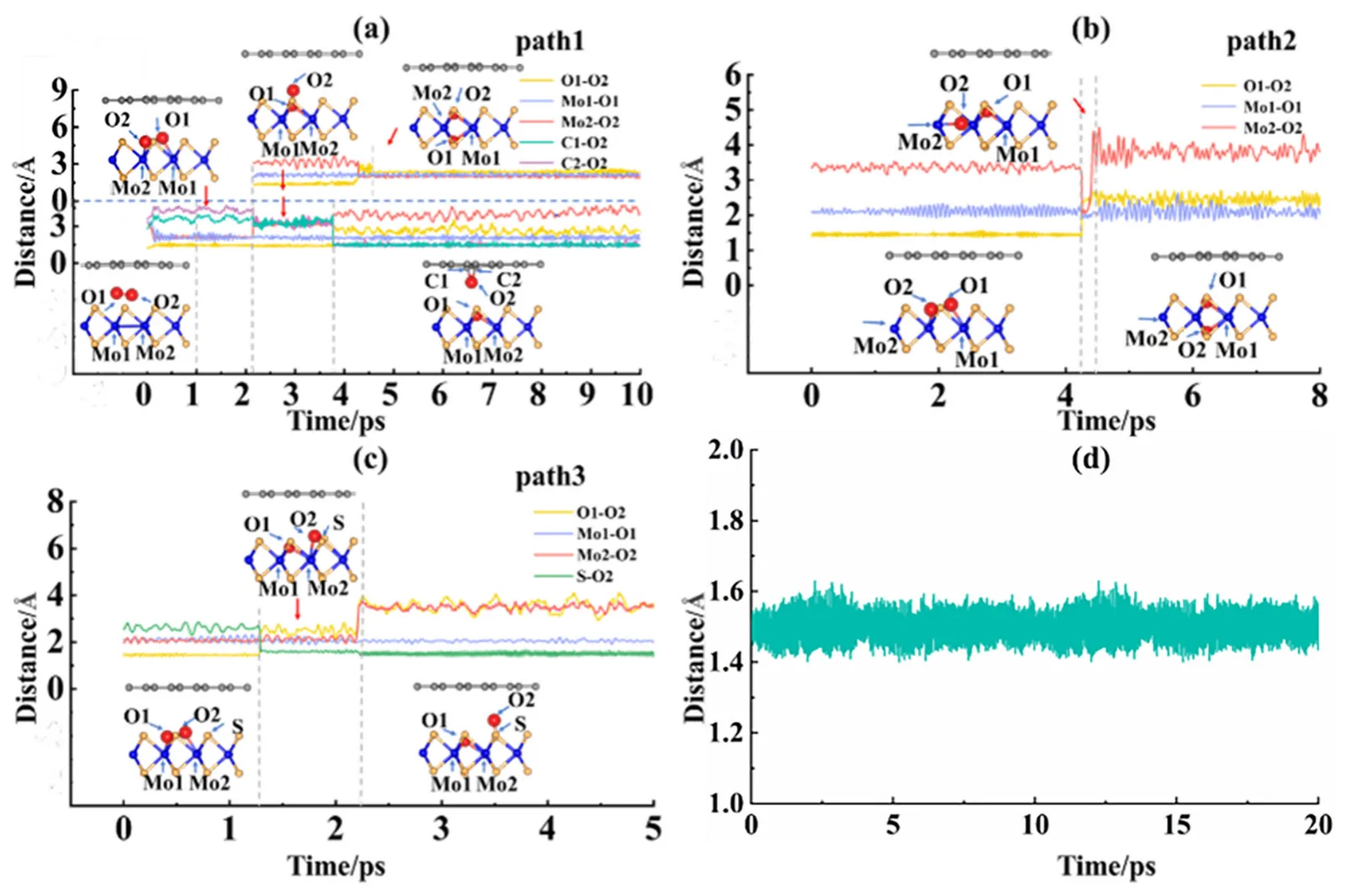
Time evolution of interatomic distances and corresponding structural changes obtained from AIMD simulations. (**a**–**c**) present the simulation results of pathway 1, pathway 2, and pathway 3 for the O_2_ reaction at the G/VS_2_ interface, respectively. (**d**) shows the variation in S-O bond length in the FS3 state. The different interatomic distances are represented as follows: O-O (orange curve), Mo-O (mauve and pink curves), C-O (purple and light green curves), and S-O (green curve).

Figure 7 presents the AIMD simulation results of O_2_ at the G/VS_2_ interface, wherein the time evolution of typical interatomic distances was displayed for the interlayer reactions. In Figure 7a, the O-O bond elongates to 1.45 Å within 1 ps, forming Mo-O bonds (~2.1 Å). Subsequently, at 2.18 ps, the Mo2-O2 bond splits, leading to two possible branching states: the formation of either an O-C bond (~1.45 Å) or an elongated O-O bond (~2.5 Å), which forms Mo-O bonds. This process supports the observation that two final states can be formed in pathway 1 (Figure 6a).

Figure 7b shows that the O-O bond can enlarge to ~2.4 Å at 4.25 ps, followed by the formation of Mo-O bonds, which eventually occupy the two S vacancies at 4.41 ps. This process is in accordance with O_2_ occupying the vacancies in pathway 2 of the G/VS_2_ interface, as shown in Figure 6b. In Figure 7c, the AIMD simulation shows that the dissociation of O_2_ can occupy a vacancy, and meanwhile form an S-O bond (~1.5 Å). This dynamical reaction behavior also agrees with the observation of an S-O bond at the G/VS interface in pathway 2. In the reaction process of pathway 3, ultimately, no desorption of SO gas was observed (Figure 7d). Overall, the simulated reaction dynamics behavior suggests that the proposed pathways for the G/VS_2_ could be feasible under certain conditions. Especially, even though pathway 2 has a high energy barrier, this reaction can still be observed at elevated temperatures.

The predicted O_2_ reaction behavior is consistent with experimental observations of sulfur-vacancy-mediated oxidation and the formation of S–O/MoO_x_ species in MoS_2_ [52,60]. Experimental studies also support oxygen incorporation into graphene and O_2_-induced passivation of disulfur vacancies in MoS_2_ [61,62]. These results provide experimental support for the defect chemistry of the G/MoS_2_ heterointerface, while our theoretical calculations further resolve the corresponding atomistic oxidation and vacancy-healing pathways.

**Figure 8:**
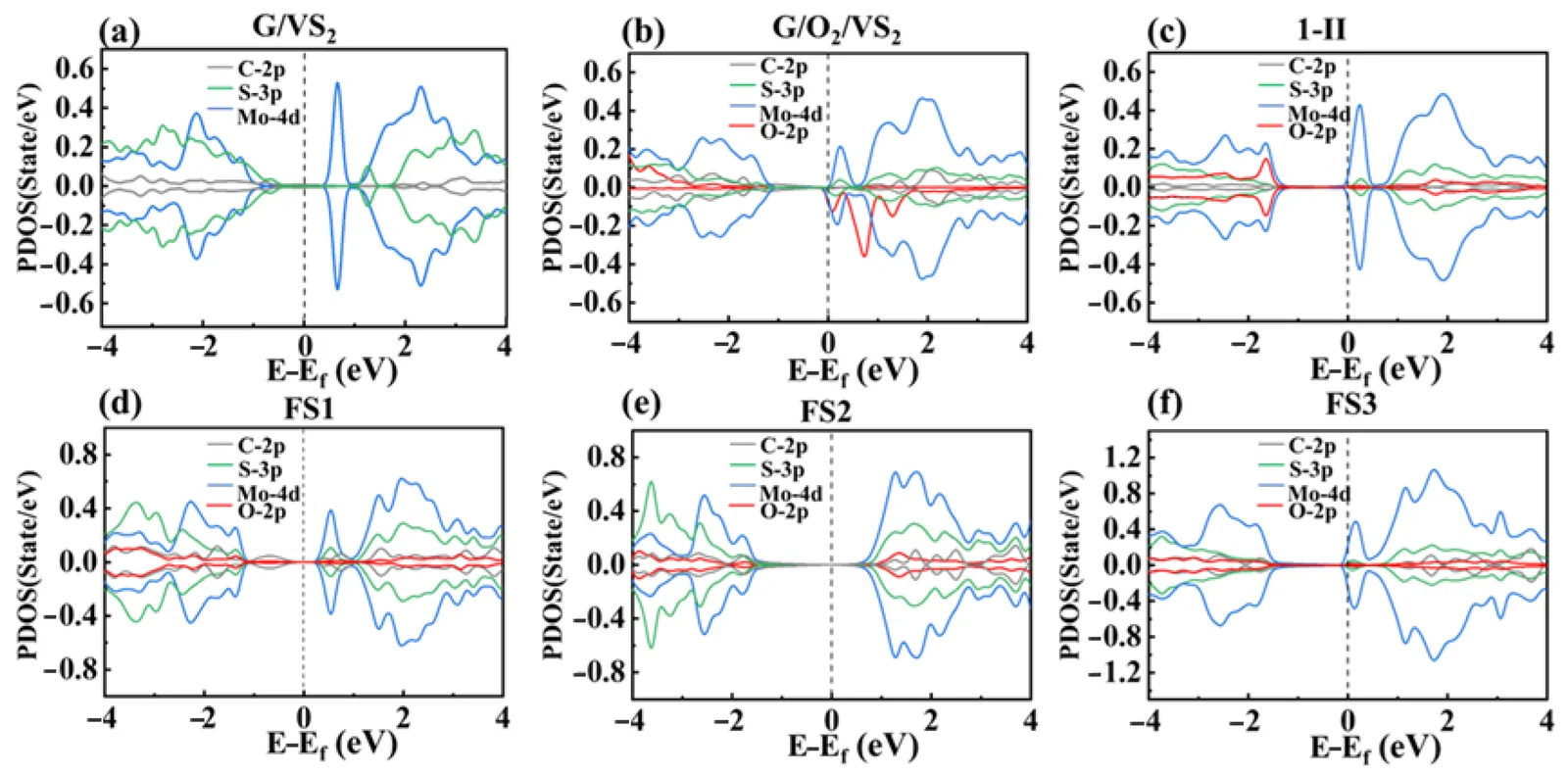
The PDOS results for the G/VS_2_ systems before and after O_2_ adsorption. (**a**) without O_2_ adsorption; (**b**) O_2_ physisorption; (**c**) O_2_ chemisorption; (**d**) FS1 state; (**e**) FS2 state; (**f**) FS3 state. Line color code: C-2p orbital, gray; S-3p orbital, green; Mo-4d orbital, blue; O-2p orbital, red. The Fermi level is set to zero and shown by a gray dashed line.

Figure 8 shows the relevant PDOS results for the G/VS_2_ system. As opposed to the simple G/VS_2_ system (Figure 8a), both physical and chemical adsorption of O_2_ (Figure 8b,c) in the interlayer causes a downward shift in defect peaks. There are obvious interactions among the 2p orbitals of O atoms, the 4d orbitals of Mo atoms, and the 3p orbitals of S atoms. For the FS1 (Figure 8d) and FS3 (Figure 8f) states, defect peaks remain observable, which is due to the residual vacancy induced by the reaction between O_2_ and G/VS_2_. Defect sites can act as active centers [63] and lead to further reactions. Meanwhile, the FS1 state exhibits a small peak near the Fermi level with the band gap increasing to 0.56 eV, attributed to the epoxide group, which alters the electronic properties of the heterojunction. Interestingly, in the FS2 state (Figure 8e), defect peaks disappear, and electronic states near the Fermi level are smoother as a result of the formation of Mo-O bonds, while the bandgap increases to 0.38 eV, thereby recovering its semiconductor nature. This indicates that O_2_ can heal the vacancy defects. In conclusion, at the G/VS_2_ interface, besides vacancy healing and graphene oxidation, partial vacancy repair may also be observed.

### 3.4. H_2_O at the Interface

We characterized the adsorption of H_2_O on the G/VS and G/VS_2_ interfaces (Figure S4a,b). The $∆E_{ads}$ for both G/VS and G/VS_2_ systems are −0.32 eV and −0.35 eV, respectively. The adsorption strengths are more stable compared to those of monolayer MoS_2_ systems [19,64,65]. The transition state searches (Figure S5a,c) show that the dissociation processes for water within the two interfaces are unfeasible in thermodynamics. The AIMD simulations (Figure S5b,d) also demonstrate that the interlayer water molecule always remains stable with no dissociation. This may be due to the insufficient vacancy size to accommodate both the OH group and the H atom simultaneously.

Next, we mainly investigated the water adsorption at the G/VMoS_2_ interface. As shown in Figure S4c, H_2_O adsorbs at the G/VMoS_2_ interface with an interlayer spacing of 3.46 Å. The $∆E_{ads}$ is −0.29 eV, which is comparable to that of VMoS_2_ without the graphene cover [19,29]. As shown in Figure 2c, H_2_O at the G/VMoS_2_ interface may proceed via two reaction pathways: Pathway 1 involves the adsorption of H_2_O at the vacancy sites of the interface, followed by a dissociation of H_2_O into an OH group and a H atom. Finally, the O atom binds to the Mo atom, while the H atom diffuses to the interface, forming S-H bonds (FS1). Pathway 2 follows a similar process, where the dissociated H atoms from the H_2_O molecule ultimately form S-H bonds at the outer surface of MoS_2_.

**Figure 9:**
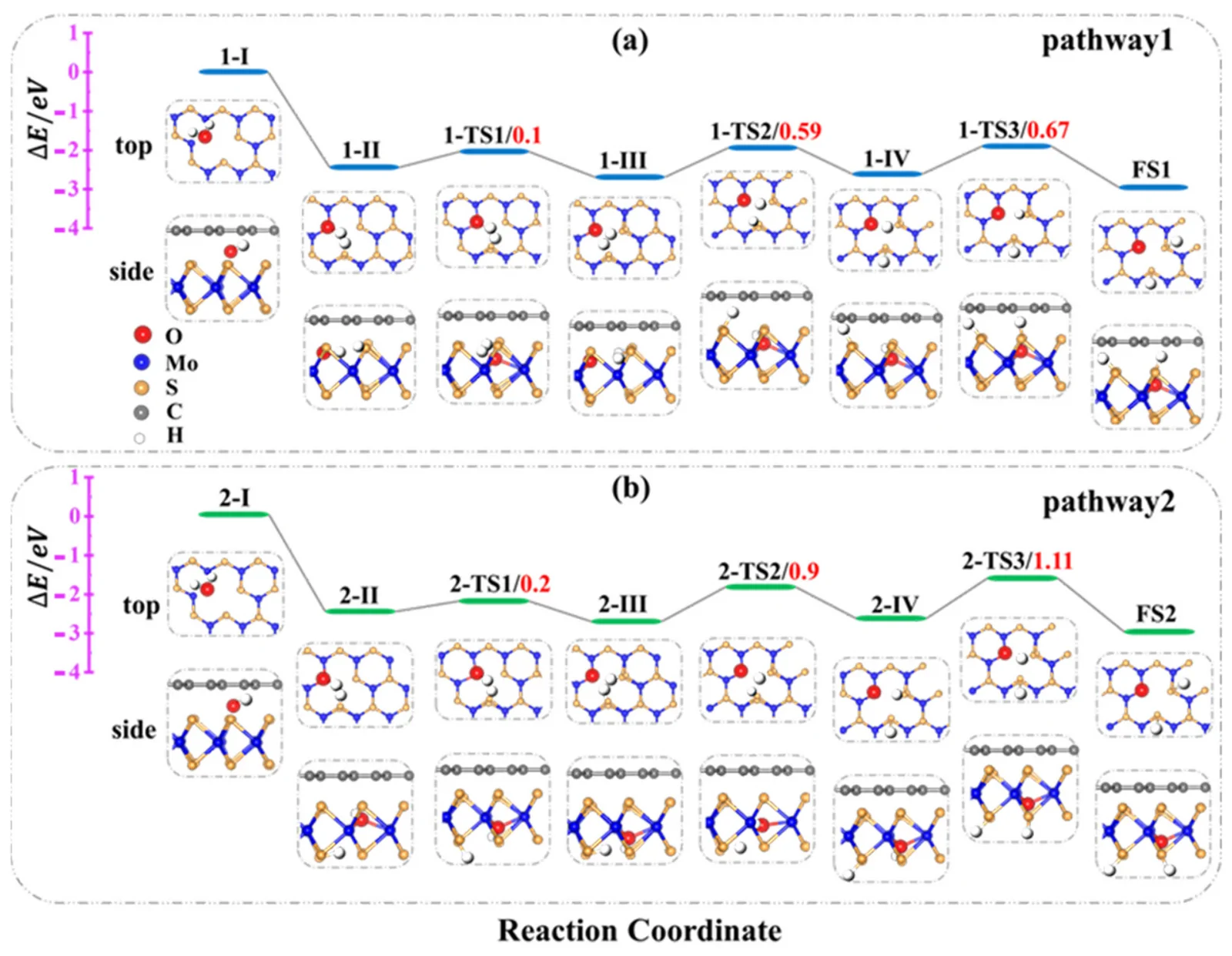
Calculated energy profiles of the reaction pathway of H_2_O at the G/VMoS_2_ interface, accompanied by the corresponding structural information (top and side views). (**a**) pathway1; (**b**) pathway2. The reaction pathway illustrates the transition state (TS) and key intermediates of the elementary reaction, with activation barrier values indicated in red.

Figure 9 presents the relevant reaction pathways and mechanisms for water at the G/VMoS_2_ interface. In pathway 1 (Figure 9a), with the H_2_O dissociating, the OH group bonds to two Mo atoms, and the H atom forms an S-H covalent bond within the interlayer (1-II). Detailed atomic bonding information is provided in Tables S7 and S8. This reaction is thermodynamically spontaneous without an energy barrier. This is attributed to the strong interaction between H_2_O and Mo atoms at the triple vacancy, which induces electron exchange and leads to the dissociation of H and O atoms [29]. This behavior demonstrates the potential water dissociation capability at the G/VMoS_2_ interface. Further calculations reveal that, under a low activation barrier (1-TS1, 0.1 eV), the H atom, originally in the OH group, dissociates and forms a new S-H covalent bond (1-III), as confirmed by the AIM result (Table S7). The H atom in the formed S-H group sequentially migrates to the Mo-Mo bridge position, with activation barriers of 0.59 eV (1-TS2) and 0.67 eV (1-TS3). Different from that of the monolayer VMoS_2_, this process has a lower activation barrier [19], demonstrating an enhanced reaction activity of water splitting.

Similarly, in pathway 2 (Figure 9b), the H_2_O molecule might dissociate, forming two S-H covalent bonds at the outer surface of MoS_2_. Compared to pathway 1, this process exhibits relatively higher activation barriers, indicating that the H_2_O dissociation reaction at the interface is less favorable. In short, the G/VMoS_2_ system exhibits promising catalytic potential toward water splitting, which might offer a possible application for engineering the heterojunction structure of graphene and MoS_2_. A Further comprehensive investigation is required and will be the subject of our future work.

Experiments have shown strong interactions between atomic hydrogen and vacancy-related sites in defective monolayer MoS_2_ [66]. In our G/VMoS_2_ interface, H species are also generated in situ through spontaneous H_2_O dissociation and subsequently form S–H bonds. Our calculations further characterize the atomistic pathways and energetic barriers associated with H generation and redistribution at the confined interface.

**Figure 10:**
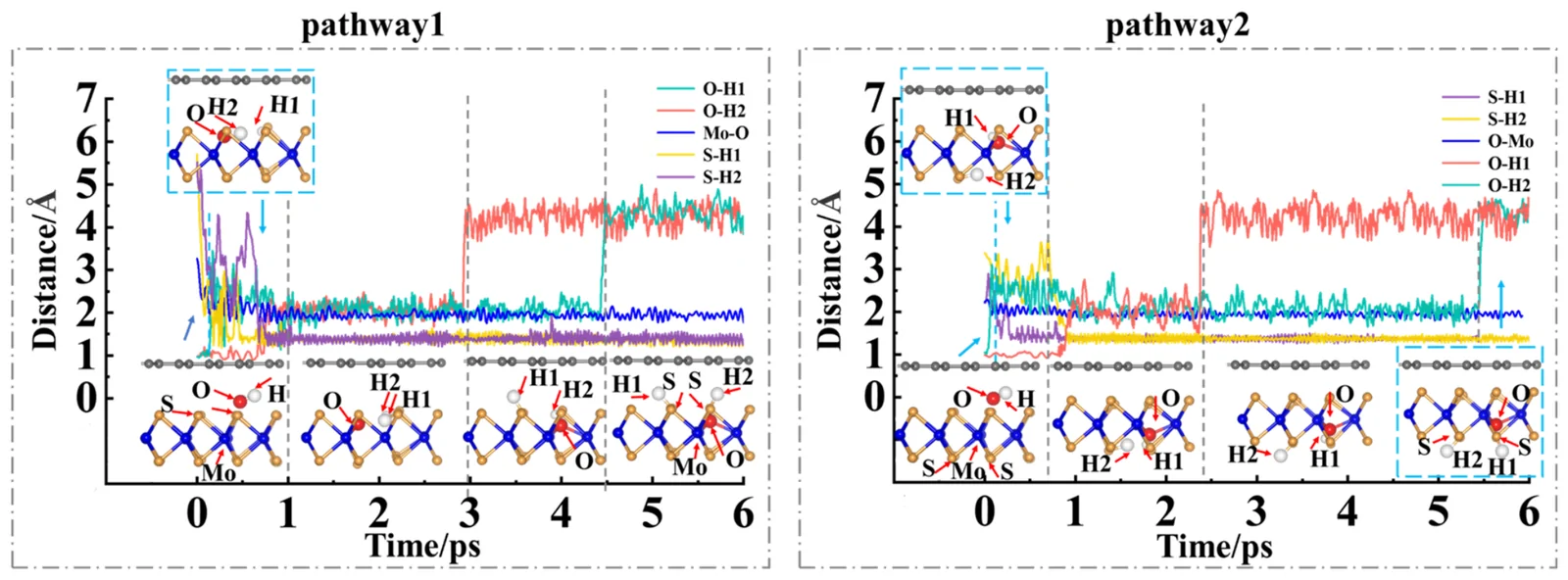
Time evolution of interfacial distances and structural changes from AIMD simulations. Simulation results of pathway 1 and pathway 2 for the reaction of H_2_O at the G/VMoS_2_ interface. The different interatomic distance systems include S-H (purple and orange curves), Mo-O (blue curve), and O-H (pink and light green).

Figure 10 presents the time evolution of interatomic distances during the reaction of H_2_O at the G/VMoS_2_ interface in the AIMD simulation, along with the corresponding structural information. It can be observed that the H_2_O molecule dissociates within 1 ps, forming Mo-O bonds (~2.1 Å). Subsequently, the H atoms form two S-H bonds (~1.35 Å), one H atom in the interlayer and the other H atom at the MoS_2_ outer surface in the G/VMoS_2_ system. The above results are consistent with the reaction pathways 1 and 2, as observed in Figure 9.

### 3.5. Interlayer Diffusion of H and O Atoms

**Figure 11:**
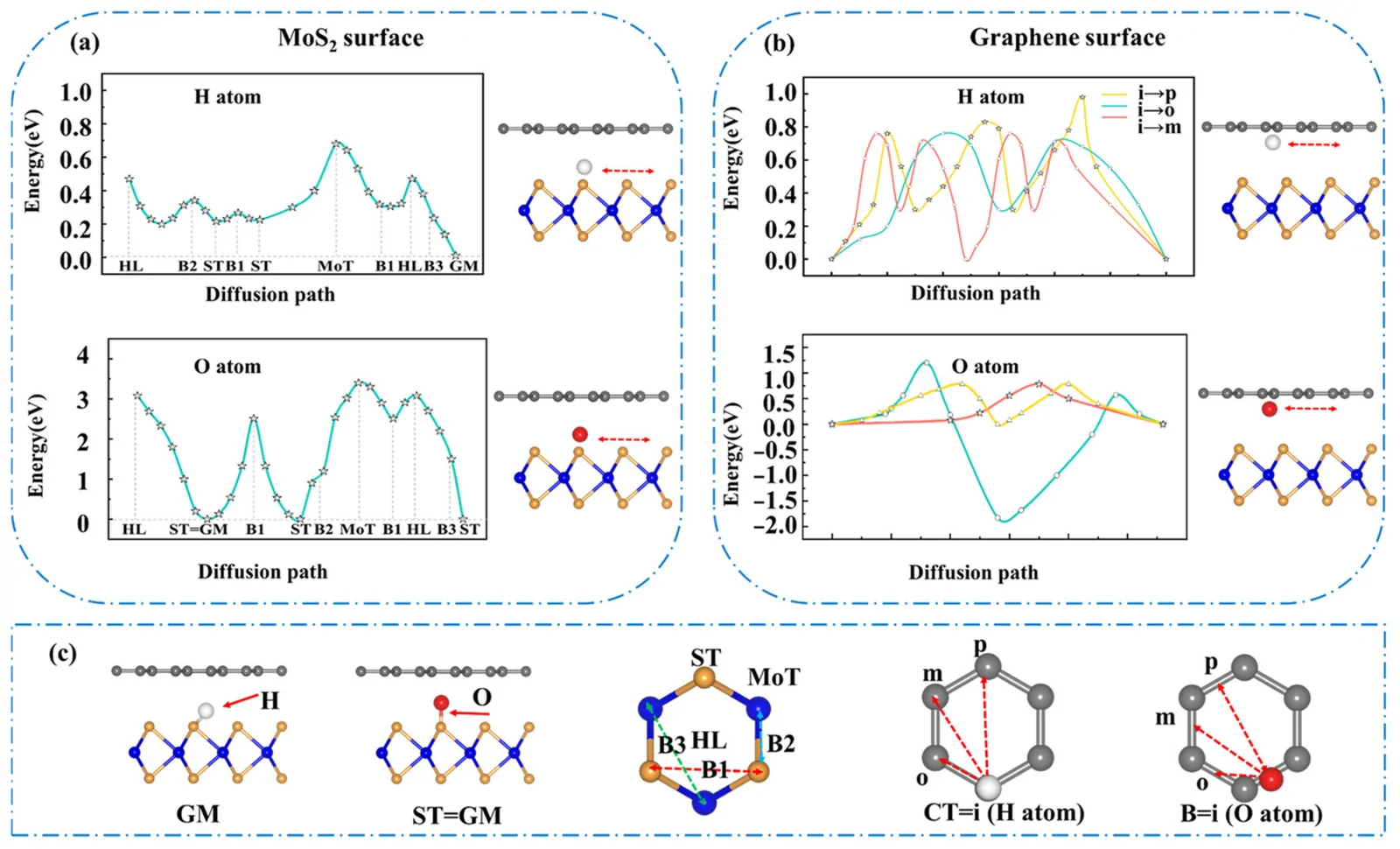
Horizontal diffusion of H and O atoms at the G/MoS_2_ interface. Diffusion paths of H and O atoms on the MoS_2_ (**a**) and graphene (**b**) surfaces. (**c**) Diffusion sites: ST, MoT (top of S and Mo atoms), B1, B2, B3 (bridge of S-S, S-Mo, and Mo-Mo bonds), HL (hexagonal center), GM (energy minimum from geometry optimization), CT (top of C atom), and B (bridge of C-C bond). Initial adsorption structures of O or H atoms on the graphene surface are labeled as i, while o, m, and p indicate diffusion positions at the ortho, meta, and para sites, respectively, relative to the i site.

In this section, we also examine the diffusion behavior of O and H atoms within the pristine G/MoS_2_ interlayer. Herein, we primarily consider the horizontal diffusion behavior of H and O atoms on the MoS_2_ and graphene surfaces. Due to the honeycomb symmetry, diffusion paths were calculated at the B, T, HL, and GM sites (bridge, top of S and Mo atoms, hollow, and geometric minimum, respectively), as shown in Figure 11c. On the MoS_2_ surface (Figure 11a), the diffusion of H atoms has low energy barriers (0.21–0.68 eV), enabling facile diffusion, in agreement with previous reports on the monolayer MoS_2_ surface [29,67]. In contrast, O atoms have higher diffusion barriers, ranging from 2.41 to 3.32 eV, indicating that O atoms are difficult to diffuse at room temperature [68]. On the graphene surface (Figure 11b), H atoms are most stably adsorbed at C-top (CT) sites [69], while O atoms prefer bridge (B) sites, forming epoxy structures [70]. The diffusion of H or O atoms occurs through migration between adjacent adsorption sites, including initial (i), meta (m), para (p), and ortho (o) positions (Figure 11c). H atoms diffuse horizontally with a barrier below 1 eV. Specifically, H atoms can diffuse freely between interlayers, implying that G/MoS_2_ heterostructures may have potential as the catalyst material for the hydrogen evolution reaction (HER). The O atom diffusion from the i site to the o or m site has a barrier of 0.78 eV. From the i site to the p site, the process occurs in two steps. Initially, the O atom dissociates from the graphene surface and forms an S-O bond with MoS_2_, requiring a diffusion barrier of 1.3 eV. Subsequently, the S-O bond breaks and the O atom diffuses to the p site on the graphene surface with a higher energy barrier of 2.4 eV. This suggests that epoxy formation may promote further oxidation. However, once the S-O bond forms, O diffusion is highly restricted.

## 4. Conclusions

In this work, a combined DFT and AIMD simulation was employed to investigate the reaction behavior of O_2_ and H_2_O at the interfaces of the G/MoS_2_ heterojunctions with different surface defects. Different mechanisms and multiple pathways have been proposed for the interlayer reactions, in which thermodynamic trends and kinetic barriers were analyzed. It is found that the pristine G/MoS_2_ interface is inert towards O_2_ and H_2_O adsorption. In contrast, the presence of defects on the MoS_2_ surface creates highly active sites for molecular adsorption and subsequent reactions.

For the G/VS interface with a single S vacancy, O_2_ can repair the vacancy and induce oxidation of the graphene surface, removing the vacancy-induced defect states, restoring the electronic structure, and altering the electronic properties. Thus, this result indicates that O_2_-induced defect healing may contribute to delaying the degradation of G/MoS_2_ in humid environments. This behavior is a dual-pathway mechanism, which is unattainable in the bare monolayer MoS_2_. For the G/VS_2_ interface with a S di-vacancy, O_2_ can repair vacancies without generating new chemical species, eliminating the vacancy-induced defect states and thereby recovering its semiconductor nature. Meanwhile, O_2_ not only induces graphene oxidation or forms an S-O bond, but also leaves a residual vacancy that could result in further oxidation, which is different from the behavior of the single S vacancy. Thus, the defect healing processes are important for controlling electronic properties in G/MoS_2_ heterostructures.

At the G/VMoS_2_ interface with a MoS_2_ triple vacancy, H_2_O can spontaneously dissociate and form S-H bonds with lower activation barriers, demonstrating its catalytic potential for water splitting. Meanwhile, the relatively facile H migration at the interface might suggest the potential relevance of G/MoS_2_ heterostructures to HER-related processes. Conversely, the diffusion of O atoms is restricted by strong S-O bonds, thus limiting further oxidation. In summary, our study provides new insights into the physical interactions, chemical reactivity, defect healing, and oxidation processes at the G/MoS_2_ heterojunction interfaces, which are important for guiding the optimization and design of G/MoS_2_-related composites or devices, such as field-effect transistors [13,71].

## Data Availability

The data that support the findings of this study are available from the corresponding author upon reasonable request.

## Supplementary Materials

The following supporting information can be downloaded at: https://www.mdpi.com/article/10.3390/nano16181173/s1, Figure S1: the optimized structure model; Figure S2: Calculated energy profiles of the reaction pathways of O_2_ (a) and H_2_O (b) at the G/MoS_2_ interface; Figure S3: The optimized structure of physisorbed O_2_ at G/VS (a) and G/VS_2_ (b) interface; Figure S4: The optimized structure of physisorbed H_2_O at G/VS (a), G/VS_2_ (b), and G/VMoS2 (c) interfaces; Figure S5: Calculated energy profiles of the reaction pathways of H_2_O at the G/VS and G/VS2 interfaces; Tables S1–S3 and Figures S6–S8: Detailed bonding information for pathway 1, 2, and 3 at the G/VS interface and the corresponding reaction structures; Tables S4–S6 and Figures S9–S11: Detailed bonding information for pathway 1, 2, and 3 at the G/VS_2_ interface and the corresponding reaction structures; Tables S7 and S8 and Figures S12 and S13: Detailed bonding information for pathway 1 and 2 at the G/VMoS_2_ interface and the corresponding reaction structures. References [44,45,72,73,74,75,76,77] are cited in the Supplementary Materials.
