# Supplementary material for: Molecular Simulation of Interfacial Chemistry of Oxygen and Water Molecules Within Defective Graphene/MoS2 Heterojunctions
Source: Nanomaterials (Basel). 2026 Sep 17;16(18):1173. doi: 10.3390/nano16181173 (PMC13610902; doi:10.3390/nano16181173)
Supplement: Supplementary file 1 [file nanomaterials-16-01173-s001.zip › nanomaterials-4507225-supplementary.pdf]

Supplementary Materials

Catalog

1. Justification of the computational methods 3

2. The optimized structure model 4

3. The reaction pathways of O_2 and H2O at the G/MoS_2 interface 5

4. The optimized structure of O_2 at G/VS and G/VS_2 interface 6

5. The optimized structure of H_2O at the interfaces 7

6. Reaction pathways and AIMD simulations of H_2O at the G/VS and G/VS_2 interfaces 8

7. Bonding information at the G/VS interface 9

8. Bonding information at the G/VS_2 interface 14

9. Bonding information at the G/VMoS_2 interface 18

1. Justification of the computational methods

To validate the rationality of the DFT computational methods, we compared the binding energy, interlayer distance, and band gap of the graphene/MoS_2 heterojunction with the previously reported results.

The calculated binding energy of the pristine G/MoS_2 system, obtained using the GGA-PBE-D3 functional, was 1.4 eV, corresponding to -28.0 meV/C atom. This value is in good agreement with the previously reported value of -28.1 meV/C atom, calculated using the GGA-PBE-D functional [1], and also agrees with -25.0 meV/C atom obtained using the GGA-PBE-D2 functional [2]. Meanwhile, the reported binding energy calculated using the optB88-vdW functional was -35.0 meV/C atom [3], while the value obtained using the B3LYP-D3(BJ) functional was -23.0 meV/C atom [4].

The optimized interlayer distance of 3.39 Å between graphene and MoS_2 in the periodic structures closely matches the experimental and theoretical values (~3.40± 0.01 Å) [5,6]. Additionally, the band gap value calculated using the HSE06 functional in our work is 13 meV, which agrees well with previous computational studies [5,7,8] and experimental observations of a small band gap (~14 meV), further supporting the accuracy of our method in electronic structure calculations.

All the comparisons confirm that the computational methods used in our work can provide reliable and reasonable calculations.

2. The optimized structure model

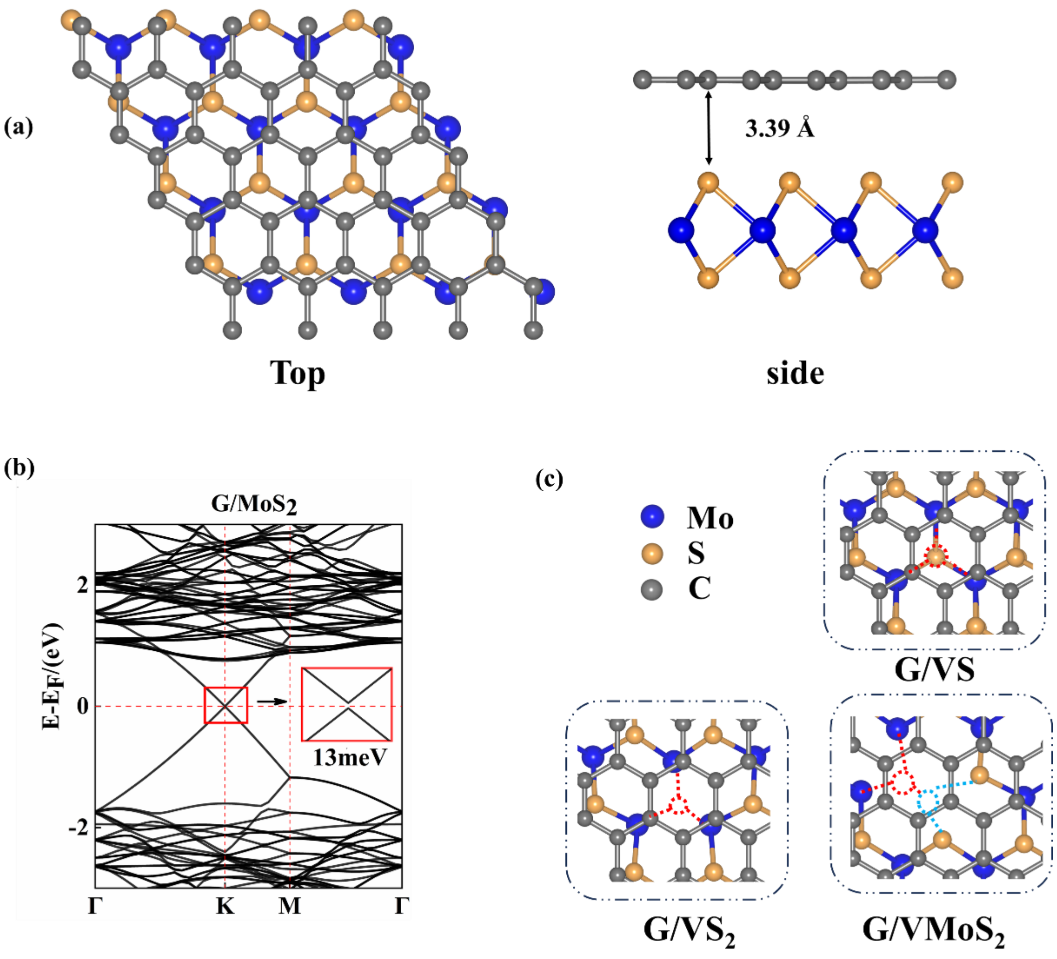

Figure S1. (a) The optimized structure of pristine G/MoS_2 heterojunction (top and side views). The black arrow indicates the distance between S and C atoms. (b) The band structure of G/MoS_2 heterojunction. (c) The optimized structures of G/VS, G/VS_2, G/$VMoS_2$. Atom color code: Mo atoms (blue), C atoms (light gray), S atoms (orange). Red and blue dashed circles denote S and Mo vacancies.

3. The reaction pathways of O_2 and H2O at the G/MoS_2 interface

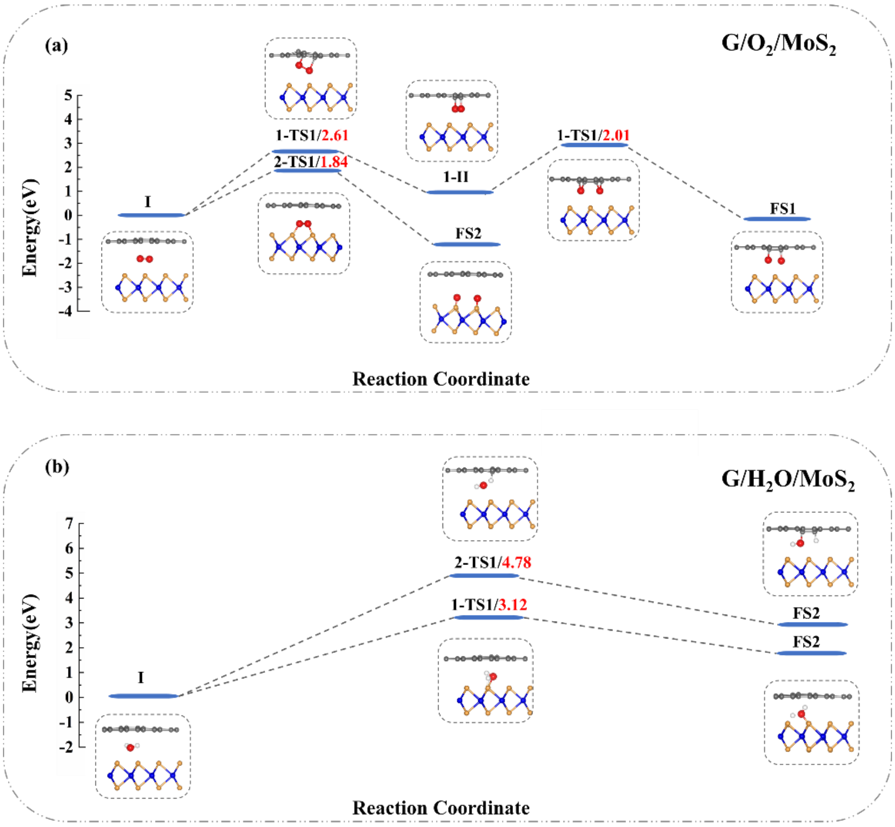

Figure S2. Calculated energy profiles of the reaction pathways of O_2 (a) and H_2O (b) at the G/MoS_2 interface, accompanied by the corresponding structural information. The reaction pathway illustrates the initial state (I), transition state (TS), final state (FS) structures, and key intermediates of the elementary reaction, with activation barrier values indicated in red.

4. The optimized structure of O_2 at G/VS and G/VS_2 interface

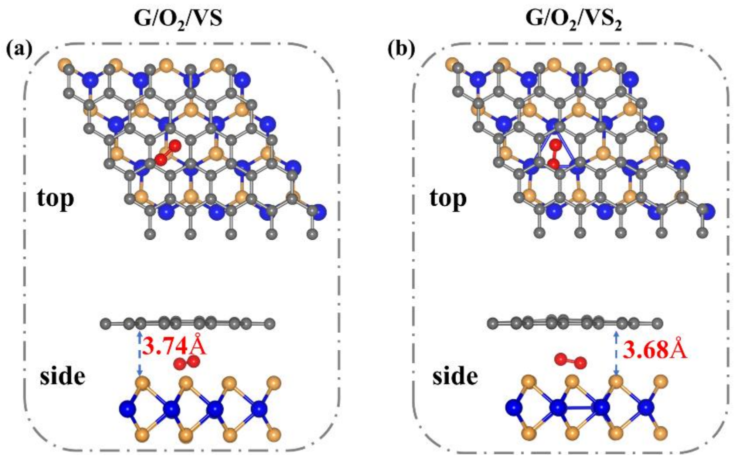

Figure S3. The optimized structure of physisorbed O_2 at G/VS (a) and G/VS_2 (b) interface (top and side views). The blue arrow indicates the distance between S and C atoms.

5. The optimized structure of H_2O at the interfaces

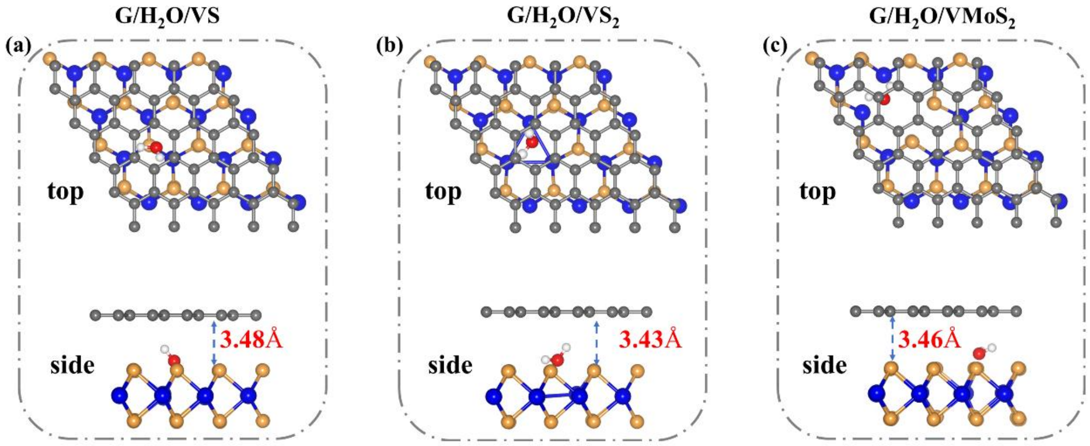

Figure S4. The optimized structure of physisorbed H_2O at G/VS (a), G/VS_2 (b), and G/$VMoS_2$(c) interfaces (top and side views). The blue arrow indicates the distance between S and C atoms.

6. Reaction pathways and AIMD simulations of H_2O at the G/VS and G/VS_2 interfaces

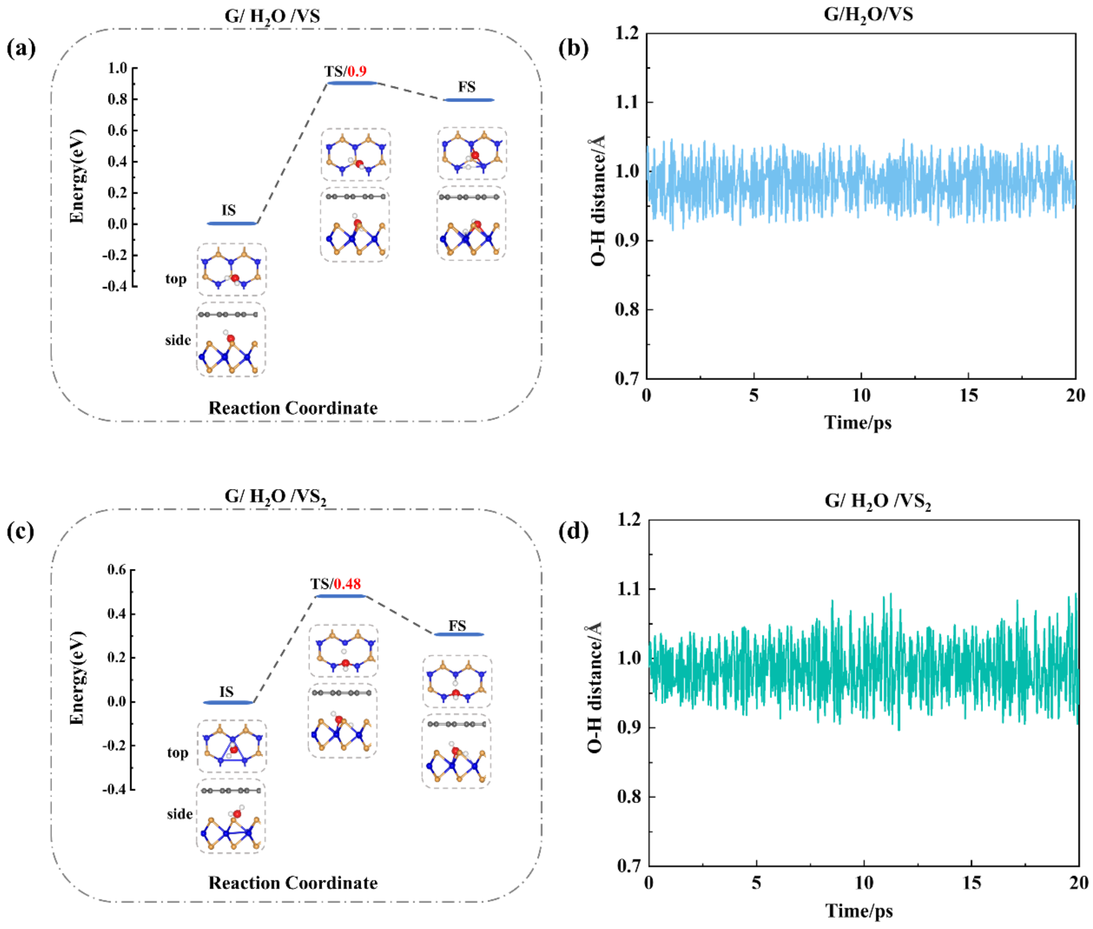

Figure S5. Calculated energy profiles of the reaction pathways of H_2O at the G/VS and G/VS_2 interfaces, accompanied by the corresponding structural information (top and side views). The reaction pathway illustrates the initial state (IS), transition state (TS), and final state (FS) structures, with activation barrier values indicated in red. The evolution of interatomic distances (O-H) of adsorbed H_2O in the G/VS (b) and G/VS_2 (d) interfaces over time, during AIMD simulations.

7. Bonding information at the G/VS interface

Table S1. Detailed bonding information for pathway 1 at the G/VS interface and simulated topological parameters at the bond critical points (BCPs), including the electron density (**ρ**), and the Laplacian value (**$\nabla^2\rho$**). The corresponding reaction structures and atomic numbers are shown in Figure S6.

State	Atom bond	ρ / a.u.	$\nabla^2\rho$ / a.u.	Atomic distance / Å
1-I	O1-O2	0.47	-0.62	1.24
1-TS1	O1-O2	0.41	-0.17	1.29
1-II	O1-O2	0.31	0.14	1.37
	Mo1-O1	0.08	0.3	2.1
	Mo2-O1	0.081	0.31	2.1
	Mo3-O1	0.08	0.3	2.1
1-TS2	O1-O2	0.087	0.32	1.86
	Mo1-O1	0.09	0.4	2.01
	Mo2-O1	0.1	0.41	2.12
	Mo3-O1	0.09	0.39	2
	C1-O2	0.047	0.13	2.16
	C1-O2	0.045	0.14	2.2
FS1	O1-O2	0.01	0.06	5.38
	C1-O2	0.23	-0.312	1.45
	C2-O2	0.24	-0.314	1.45
	Mo1-O1	0.088	0.39	2.1
	Mo2-O1	0.087	0.41	2.12
	Mo3-O1	0.09	0.4	2.1

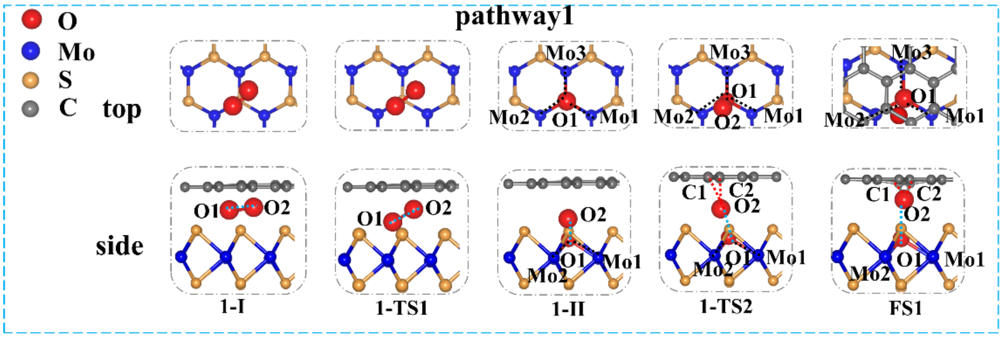

Figure S6. The reaction structures and atomic numbers of adsorbed O_2 in reaction pathway 1 at the interface of the G/VS system.

Table S2. Detailed bonding information for pathway 2 at the G/VS interface and simulated topological parameters at the BCPs, including the electron density (***ρ***), and the Laplacian value (**$\nabla^2\rho$**). The corresponding reaction structures and atomic numbers are shown in Figure S7.

State	Atom bond	***ρ*** / a.u.	**$\nabla^2\rho$** / a.u.	Atomic distance / Å
2-I	O1-O2	0.31	0.14	1.37
	Mo1-O1	0.08	0.3	2.1
	Mo2-O1	0.081	0.31	2.1
	Mo3-O1	0.08	0.3	2.1
2-TS1	O1-O2	0.27	0.13	1.45
	Mo1-O1	0.05	0.22	2.35
	Mo2-O1	0.1	0.41	2.01
	Mo3-O1	0.1	0.42	2.01
	Mo3-O2	0.04	0.143	2.5
2-II	O1-O2	0.27	0.03	1.43
	Mo1-O1	0.09	0.32	2.1
	Mo2-O1	0.09	0.32	2.1
	Mo3- O2	0.1	0.3	2.07
2-TS2	O1-O2	0.08	0.29	1.92
	Mo1-O1	0.1	0.3	2.01
	Mo2-O1	0.09	0.32	2.1
	Mo3-O2	0.11	0.38	2
	S1-O2	0.13	0.11	1.89
2-III	O1-O2	/	/	2.45
	Mo1-O1	0.11	0.38	2.1
	Mo2-O1	0.1	0.35	2
	Mo3-O1	0.11	0.37	2
	Mo3-O2	0.08	0.29	2.15
	S1-O2	0.23	0.11	1.58
2-TS3	O1-O2	/	/	2.69
	Mo1-O1	0.11	0.38	2
	Mo2-O1	0.1	0.35	2
	Mo3-O1	0.11	0.37	2
	Mo3-O2	/	/	2.74
	S1-O2	0.24	0.2	1.54
FS2	O1-O2	/	/	3.53
	S1-O2	0.27	0.56	1.48

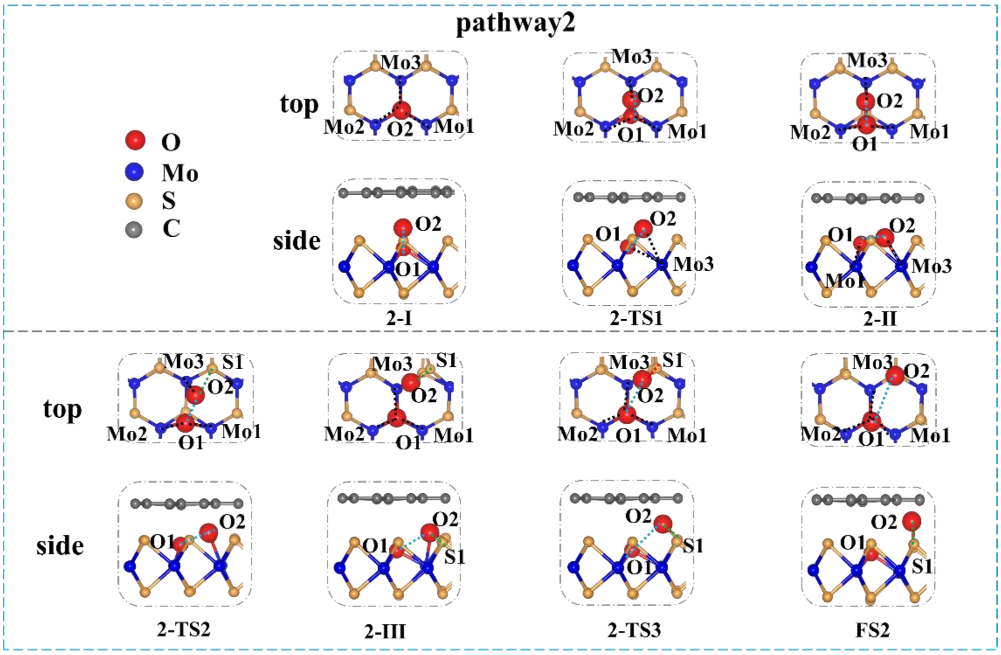

Figure S7. The reaction structures and atomic numbers of adsorbed O_2 in reaction pathway 2 at the interface of the G/VS system.

Table S3. Detailed bonding information for pathway 3 at the G/VS interface and simulated topological parameters at BCPs, including the electron density (***ρ***), and the Laplacian value (**∇²*ρ***), are provided. The corresponding reaction structures and atomic numbers are shown in Figure S8.

State	Atom bond	***ρ*** / a.u.	**∇²*ρ*** / a.u.	Atomic distance / Å
3-I	O1-O2	0.27	0.03	1.43
	Mo1-O1	0.09	0.32	2.1
	Mo2-O1	0.09	0.3	2.1
	Mo3- O2	0.1	0.3	2.07
3-TS1	Mo1-O1	0.09	0.32	2.1
	Mo2-O1	0.09	0.32	2.1
	Mo3- O2	0.1	0.3	2.07
	Mo1-O2	0.14	0.57	2.16
	Mo2-O2	0.12	0.47	2
	Mo4-O2	0.035	0.078	2.6
3-II	O1-O2	0.047	0.25	2.2
	Mo1-O1	0.13	0.41	2
	Mo2-O1	0.07	0.25	2.2
	Mo3-O1	0.075	0.21	2.2
	Mo1-O2	0.14	0.54	2
	Mo2-O2	0.15	0.54	1.9
	Mo4-O2	0.14	0.5	1.9
3-TS2	O1-O2	/	/	2.35
	Mo1-O1	0.12	0.41	2
	Mo2-O1	0.09	0.29	2.1
	Mo3-O1	0.09	0.29	2.1
	Mo1-O2	0.13	0.5	1.9
	Mo2-O2	0.15	0.56	2
	Mo4-O2	0.08	0.26	2.17
	S2-O2	0.06	0.18	2.3
3-III	O1-O2	/	/	2.6
	Mo1-O1	0.099	0.35	2.01
	Mo2- O1	0.11	0.39	2.02
	Mo3- O1	0.11	0.39	2.02
	Mo2- O2	0.09	0.29	2.14
	S2-O2	0.18	0.019	1.68
3-TS3	O1-O2	/	/	4
	S2-O2	0.25	0.33	1.5
	Mo2-O2	0.023	0.094	2.7
3-IV	O1-O2	/	/	3.42
	S2-O2	0.23	0.14	1.57
	Mo2-O2	0.05	0.16	2.34
3-TS4	O1-O2	/	/	3.8

	S2-O2	0.24	0.26	1.54
	Mo2-O2	0.028	0.086	2.69
FS3	S2-O2	0.26	0.487	1.45

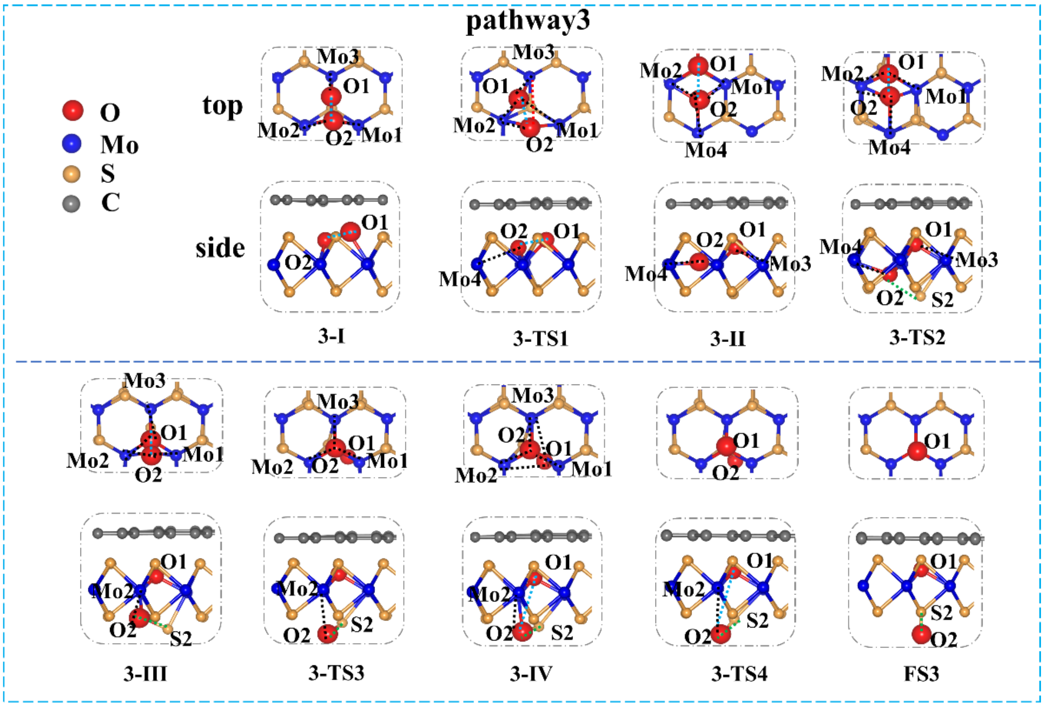

Figure S8. The reaction structures and atomic numbers of adsorbed O_2 in reaction pathway 3 at the interface of the G/VS system.

8. Bonding information at the G/VS_2 interface

Table S4. Detailed bonding information for pathway 1 at the G/VS_2 interface and simulated topological parameters at the BCPs, including the electron density (***ρ***), and the Laplacian value (**∇²*ρ***). The corresponding reaction structures and atomic numbers are shown in Figure S9.

State	Atom bond	ρ / a.u.	$\nabla^2\rho$ / a.u.	Atomic distance / Å
1-I	O1-O2	0.47	-0.613	1.24
	Mo1-O2	0.017	0.039	3.3
1-TS1	O1-O2	0.43	-0.3	1.27
	Mo1-O2	0.027	0.073	2.7
1-II/2-I	O1-O2	0.28	0.018	1.43
	Mo1-O2	0.10	0.32	2.07
	Mo2-O1	0.09	0.33	2.1
	Mo3-O1	0.09	0.33	2.1
1-TS2	O1-O2	0.26	0.12	1.45
	Mo1-O2	0.05	0.17	2.4
	Mo1-O1	0.11	0.45	2.
	Mo2-O1	0.1	0.45	2
1-III	O1-O2	0.29	0.145	1.4
	Mo1-O1	0.09	0.37	2.09
	Mo2-O1	0.09	0.37	2.1
	Mo3-O1	0.09	0.36	2.09
1-TS3-1	O1-O2	0.07	0.26	2
	O2-C1	0.05	0.16	1.94
	O2-C2	0.07	0.15	1.9
1-TS3-2	O1-O2	0.24	0.28	1.48
	Mo1-O1	0.08	0.38	2.1
	Mo1-O2	0.06	0.25	2.3
	Mo2-O1	0.12	0.58	2
	Mo3-O1	0.12	0.58	2
FS1	O1-O2	0.01	0.07	2.62
	C1-O2	0.24	-0.32	1.45
	C2-O2	0.24	-0.31	1.45
FS2	O1-O2	0.04	0.19	2.4
	Mo1-O1	0.1	0.34	2.06
	Mo1-O2	0.1	0.34	2.06
	Mo2-O1	0.1	0.34	2.06
	Mo2-O2	0.1	0.33	2.06
	Mo3-O1	0.1	0.34	2.06
	Mo3-O2	0.1	0.33	2.06

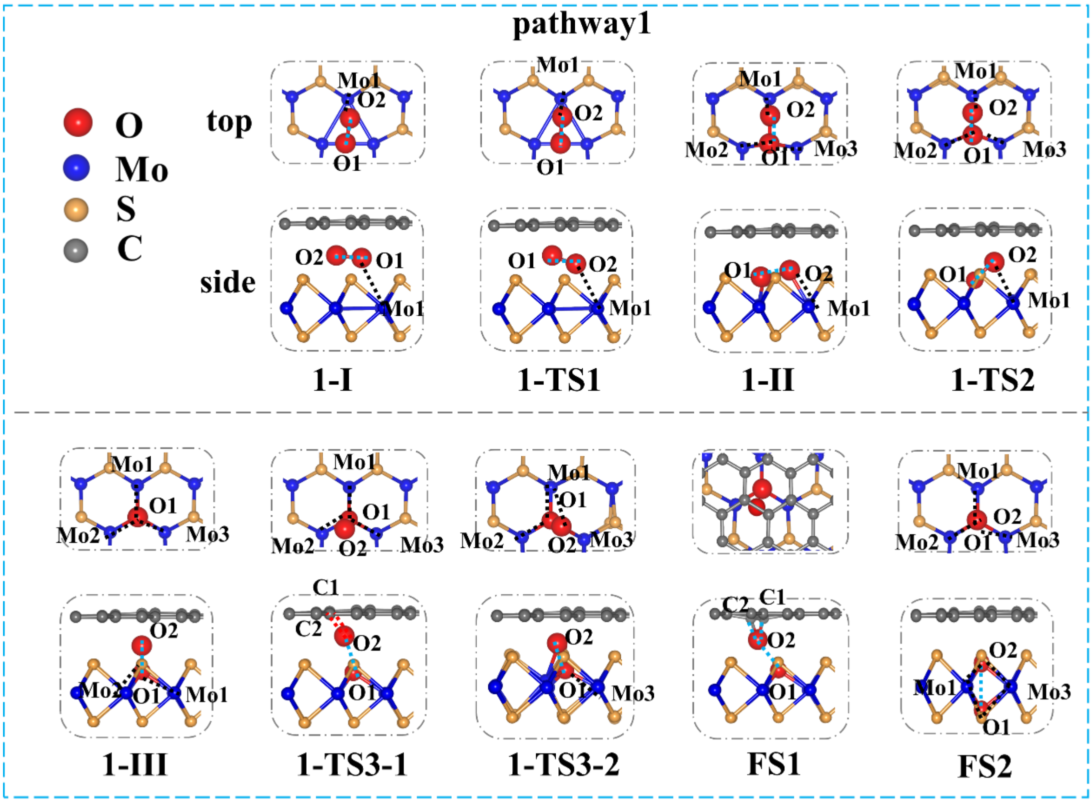

Figure S9. The reaction structures and atomic numbers of adsorbed O_2 in reaction pathway 1 at the interface of the G/VS_2.

Table S5. Detailed bonding information for pathway 2 at the G/VS_2 interface and simulated topological parameters at the BCPs, including the electron density (ρ), and the Laplacian value ($\nabla^2\rho$). The corresponding reaction structures and atomic numbers are shown in Figure S10.

State	Atom bond	ρ / a.u.	$\nabla^2\rho$ / a.u.	Atomic distance / Å
2-I	O1-O2	0.28	0.018	1.43
	Mo1-O2	0.10	0.32	2.07
	Mo2-O1	0.09	0.33	2.1
	Mo3-O1	0.09	0.33	2.1
2-TS1	O1-O2	0.11	0.37	1.8
	Mo1-O1	0.12	0.39	2
	Mo2-O2	0.15	0.63	1.9
	Mo3-O2	0.16	0.63	1.9
2-II	O1-O2	/	/	2.4
	Mo1-O1	0.12	0.41	2.1
	Mo2-O1	0.08	0.26	2.1
	Mo3-O1	0.085	0.262	2.1
	Mo4-O2	0.11	0.38	2
2-TS2	O1-O2	/	/	2.42
	Mo1-O1	0.08	0.27	2.1
	Mo2-O1	0.07	0.26	2.1
	Mo3-O1	0.12	0.44	2.1
	Mo4-O2	0.09	0.31	2.11
FS2	O1-O2	0.04	0.19	2.4
	Mo1-O1	0.1	0.34	2.06
	Mo1-O2	0.1	0.34	2.06
	Mo2-O1	0.1	0.34	2.06
	Mo2-O2	0.1	0.33	2.06
	Mo3-O1	0.1	0.34	2.06
	Mo3-O2	0.1	0.33	2.06

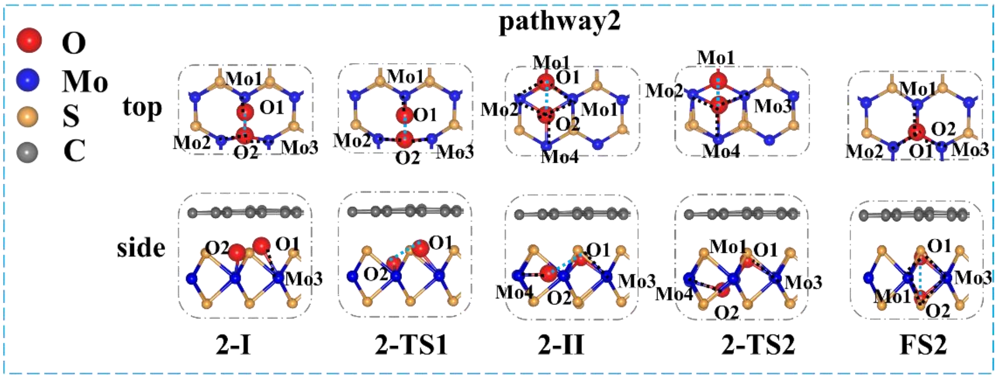

Figure S10. The reaction structures and atomic numbers of adsorbed O_2 in reaction pathway 2 at the interface of the G/VS_2.

Table S6. Detailed bonding information for pathway 3 at the G/VS_2 interface and simulated topological parameters at the BCPs, including the electron density (ρ), and the Laplacian value ($\nabla^2\rho$). The corresponding reaction structures and atomic numbers are shown in Figure S11.

State	Atom bond	ρ / a.u.	$\nabla^2\rho$ / a.u.	Atomic distance / Å
3-I	O1-O2	0.28	0.018	1.43
	Mo1-O2	0.10	0.32	2.07
	Mo2-O1	0.09	0.33	2.1
	Mo3-O1	0.09	0.33	2.1
3-TS1	O1-O2	0.073	0.28	1.95
	Mo1-O1	0.03	0.064	2.76
	Mo1-O2	0.1	0.39	2.07
	S-O2	0.14	0.06	1.84
	Mo2-O1	0.11	0.4	2.1
	Mo3-O1	0.11	0.36	2.1
3-II	O1-O2	0.11	0.39	2.44
	S-O2	0.22	0.11	1.59
	Mo1-O2	0.089	0.29	2.13
3-TS2	O1-O2	/	/	2.66
	S-O2	0.24	0.23	1.53
	Mo1-O2	/	/	2.79
FS3	O1-O2	0.107	0.36	3.51
	S-O2	0.27	0.57	1.45

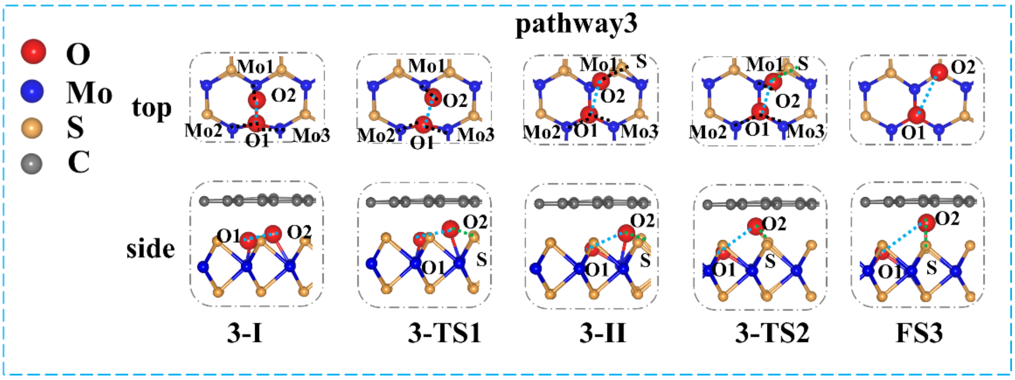

Figure S11. The reaction structures and atomic numbers of adsorbed O_2 in reaction pathway 3 at the interface of the G/VS_2.

9. Bonding information at the G/VMoS$_2$ interface

Table S7. The detailed bonding information for pathway 1 at the G/VMoS$_2$ interface and simulated topological parameters at the BCPs, including the electron density (**ρ**), and the Laplacian value (**$\nabla^2\rho$**). The corresponding reaction structures and atomic numbers are shown in Figure S12.

State	Atom bond	ρ / a.u.	$\nabla^2\rho$ / a.u.	Atomic distance / Å
1-I	O-H1	0.35	-0.22	0.98
	O-H2	0.34	-0.22	0.98
1-II	O-H1	0.28	-0.147	0.99
	O-H2	/	/	2.78
	Mo1-O	0.11	0.48	2
	Mo2-O	0.11	0.47	2
	S1-H2	0.2	-0.71	1.38
1-TS1	O-H1	0.3	-0.18	1
	O-H2	/	/	2.47
	S1-H2	0.19	-0.68	1.39
	S2-H1	0.018	-0.058	2.46
	O-Mo1	0.11	0.49	2
	O-Mo2	0.12	0.5	2
1-III	O-H1	0.025	0.1	2.1
	O-H2	/	/	1.99
	S1-H2	0.21	-0.77	1.37
	S2-H1	0.21	-0.76	1.38
1-TS2	S1-H2	0.2	-0.74	1.36
	S2-H1	0.2	-0.73	1.37
1-VI	S1-H2	0.21	-0.76	1.37
	S2-H1	0.2	-0.72	.37
1-TS3	S1-H2	0.2	-0.69	1.37
	S2-H1	0.2	-0.76	1.36
FS1	S1-H2	0.21	-0.71	1.37
	S2-H1	0.21	-0.73	1.37

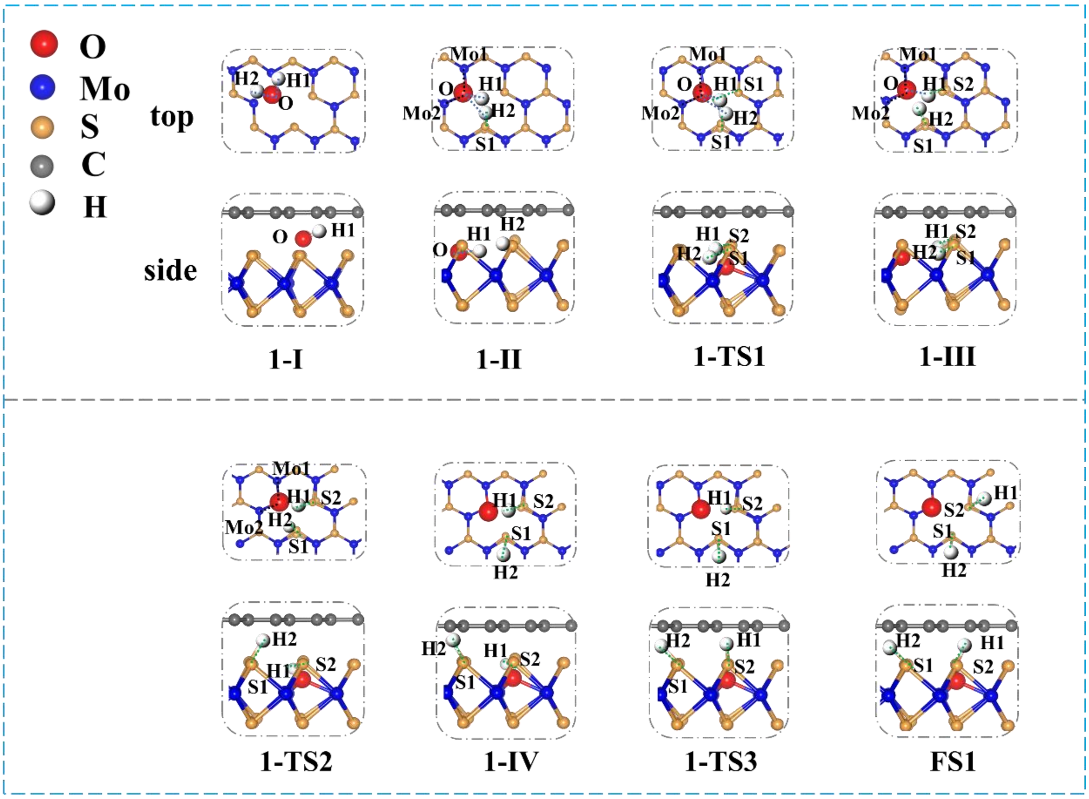

Figure S12. The reaction structures and atomic numbers of adsorbed H_2O in reaction pathway 1 at the interface of the G/$VMoS_2$.

Table S8. Detailed bonding information for pathway 2 at the G/VMoS_2 interface and simulated topological parameters at the BCPs, including the electron density (ρ), and the Laplacian value ($\nabla^2\rho$). The corresponding reaction structures and atomic numbers are shown in Figure S13.

State	Atom bond	ρ / a.u.	$\nabla^2\rho$ / a.u.	Atomic distance / Å
2-I	O-H1	0.35	-0.22	0.98
	O-H2	0.34	-0.22	0.98
2-II	O-H1	0.27	-0.13	0.98
	O-H2	/	/	2.66
	Mo1-O	0.1	0.45	2.13
	Mo2-O	0.11	0.44	2.12
	S1-H2	0.21	-0.72	1.37
2-TS1	O-H1	0.28	-0.17	1.24
	O-H2	/	/	2.4
	S1-H2	0.21	-0.74	1.37
	S2-H1	0.28	-0.11	1.65
	O-Mo1	0.11	0.44	2
	O-Mo2	0.1	0.44	2
2-III	O-H1	0.02	0.09	2.02
	O-H2	/	/	2.17
	S1-H2	0.2	-0.71	1.37
	S2-H1	0.21	-0.78	1.36
2-TS2	S1-H2	0.2	-0.74	1.36
	S2-H1	0.21	-0.74	1.37
2-VI	S1-H2	0.21	-0.75	1.37
	S2-H1	0.21	-0.79	1.37
2-TS3	S1-H2	0.2	-0.74	1.37
	S2-H1	0.21	-0.75	1.37
FS2	S1-H2	0.21	-0.73	1.37
	S2-H1	0.21	-0.76	1.37

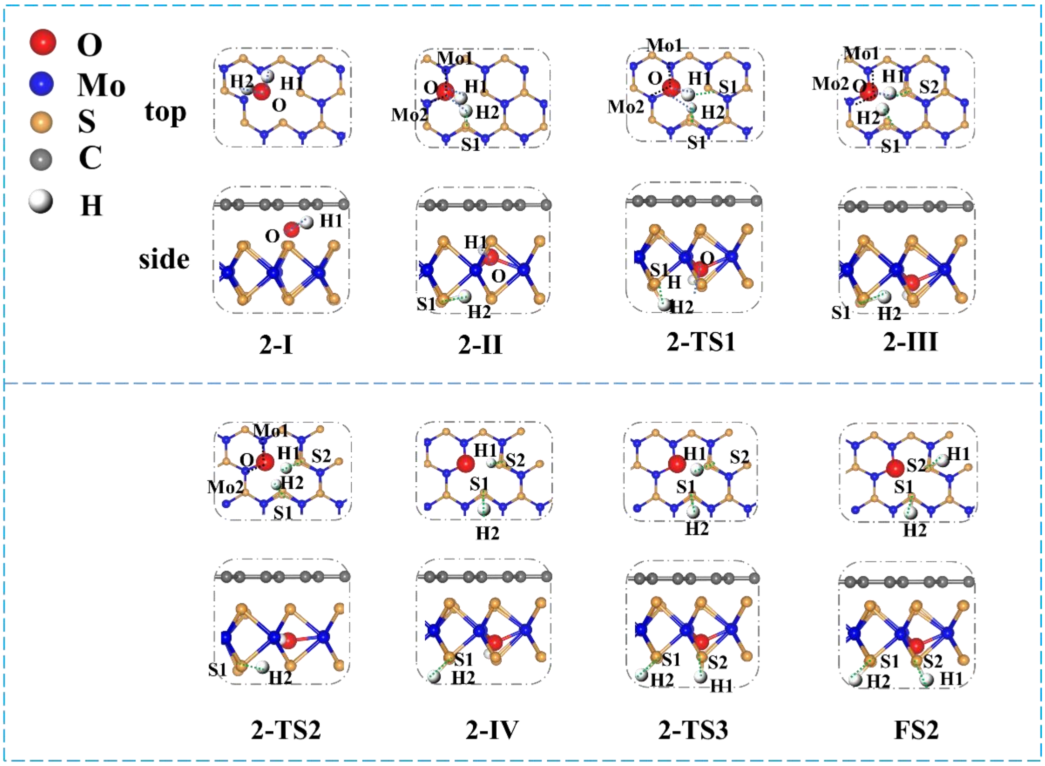

Figur S13. The reaction structures and atomic numbers of adsorbed H_2O in reaction pathway 2 at the interface of the G/VMoS_2

References

1. Hossain, M. T.; Rahman, M. A. A first principle study of the structural, electronic, and temperature-dependent thermodynamic properties of graphene/MoS_2 heterostructure. *J. Mol. Model*. **2020**, *26*, 40.
2. Phuc, H. V.; Hieu, N. N.; Hoi, B. D.; Phuong, L. T. T.; Nguyen, C. V. First principle study on the electronic properties and Schottky contact of graphene adsorbed on MoS_2 monolayer under applied out-plane strain. *Surf. Sci.* **2018**, *668*, 23-28.
3. Ghobadi, N. Normal compressive strain-induced modulation of electronic and mechanical properties of multilayer MoS_2 and Graphene/MoS_2 heterostructure: A first-principles study. *Physica E* **2019**, *111*, 158-166.
4. Xu, C.; Ding, Y.; Wang, S.; Cao, S. The van der Waals interaction and absorption and electron circular dichroism spectra of two-dimensional bilayer stacked structures. *Spectrochim. Acta, Part A.* **2023**, *303*, 123182.
5. Pierucci, D.; Henck, H.; Avila, J.; Balan, A.; Naylor, C. H.; Patriarche, G.; Dappe, Y. J.; Silly, M. G.; Sirotti, F.; Johnson, A. T. C.; Asensio, M. C.; Ouerghi, A. Band Alignment and Minigaps in Monolayer MoS_2-Graphene van der Waals Heterostructures. *Nano Lett.* **2016**, *16*, 4054-4061.
6. Kim, S.; Lee, C.; Lim, Y. S.; Shim, J.-H. Investigation for Thermoelectric Properties of the MoS_2 Monolayer–Graphene Heterostructure: Density Functional Theory Calculations and Electrical Transport Measurements. *ACS Omega* **2021**, *6*, 278-283.
7. Yu, L.; Lee, Y.-H.; Ling, X.; Santos, E. J. G.; Shin, Y. C.; Lin, Y.; Dubey, M.; Kaxiras, E.; Kong, J.; Wang, H.; Palacios, T. Graphene/MoS_2 Hybrid Technology for Large-Scale Two-Dimensional Electronics. *Nano Lett.* **2014**, *14*, 3055-3063.
8. Fu, S.; Wang, D.; Ma, Z.; Liu, G.; Zhu, X.; Yan, M.; Fu, Y. The first-principles study on the halogen-doped graphene/MoS_2 heterojunction. *Solid State Commun.* **2021**, *334-335*, 114366.
